# Unwrapping the mirror tracing task

**DOI:** 10.3758/s13428-025-02845-6

**Published:** 2026-03-05

**Authors:** Pablo F. Garrido, Anne Cecilie Sjøli Bråthen, Emilie Sogn Falch, Jonas Kransberg, Anders M. Fjell, Øystein Sørensen, Kristine B. Walhovd

**Affiliations:** 1 Center for Lifespan Changes in Brain and Cognition, Department of Psychology, University of Oslo, POB 1094 Blindern, 0317 Oslo, Norway; 2 Department of Physics, University of Oslo, Oslo, Norway; 3 Computational Radiology and Artificial Intelligence, Department of Radiology and Nuclear Medicine, Oslo University Hospital, Oslo, Norway

**Keywords:** Mirror tracing task, Time series analysis, Polar coordinates, Data clustering, Aging

## Abstract

The Mirror Tracing Task (MTT) is a method used to study visuomotor skills learning. It is traditionally evaluated by counting the number of times a person draws outside of the borders of a figure, typically a star, while looking at its mirror reflection. While insightful for overall performance, this metric lacks a precise analysis of the tracing, such as details on errors in specific regions. We propose a new MTT analysis method that studies the drawing as a function of the angle around the figure’s center. Two new variables are introduced: residuals, which measure deviation from the ideal drawing, and density, which measures how often a specific path is retraced. These variables are defined per angle or region, allowing a more detailed analysis, highlighting the most challenging parts of the drawing for each person, and enabling comparison across trials or finding common patterns between individuals. We applied this approach to the first MTT trial of 210 participants using age as a variable of interest. Residuals and density were summarized as a single value and compared with the traditional approach, providing similar results. When analyzed as a function of the angle, these variables enabled the identification of specific regions of the star where the errors are age-sensitive. Additionally, a time series-like approach enabled us to cluster drawings and quantify their similarity. The code used for this new method has been made openly accessible to make it easier for its applications in new research or the reanalysis of previous projects.

**Supplementary Information:**

The online version contains supplementary material available at 10.3758/s13428-025-02845-6.

## Introduction

The Mirror Tracing Task (MTT) is a neuropsychological test that measures visuomotor skills and cognitive flexibility. It is particularly known for measuring procedural memory and motor learning when administered over multiple trials and is widely used for clinical assessments and experimental research. This test had special relevance in the study of the amnesic patient H. M. in the early 1950 s (Corkin, 2002; Milner et al., 1998; Scoville & Milner, 1957), and it is broadly used across research fields, including development, aging, neuropsychiatric and neurodegenerative diseases (Dumel et al., 2015; Finn et al., 2016; Gabrieli et al., 1993; Harrison et al., 2014; Julius & Adi-Japha, 2016; Lemieux et al., 2014; Miall et al., 2021; Rasch et al., 2009; Rawn & Keller, 2023; Renna et al., 2018; Romanowska & Best, 2023; Schloss & Haaga, 2011; Veilleux et al., 2019).

In short, the task measures a person’s ability to draw within the lines of a double-contour star without crossing its borders. The task is especially demanding as it requires tracing the star while relying on its inverted reflection in a mirror for visual input. A person trying the test for the first time will find the changing of directions cognitively challenging due to previously overlearned conflicting responses (Lemieux et al., 2014). The traditional MTT quantification measures include Time (total time spent on the task) and Errors (number of times the drawn trace crosses any of the star’s two borders), which offers valuable information regarding a person’s motor skills and learning abilities, including visuomotor adaptation, control or inhibition of overlearned responses, procedural learning, error correction, and long-term retention of acquired skills, among others (Alrubaye et al., 2024; Brosseau et al., 2007; Corkin, 2002; Dumel et al., 2015; Finn et al., 2016; Gabrieli et al., 1993; Lemieux et al., 2014; Mantua et al., 2016; Miall et al., 2021; Milner et al., 1998; Nissen et al., 2006; Rouleau, Décary, et al., 2002a, b; Rouleau, Salmon, et al., 2002a, b; Vicari et al., 2005; Waldrop et al., 2001).

However, while timing and simply counting the number of errors on MTT have proven sensitive to a series of conditions, these are limited measures relative to what can be gained from the task. The traditional approach provides only an overview of the entire tracing. When comparing drawings, for example, it tells us that there may be differences between them, but not exactly where or how relevant those differences are. As the figures used in the task combine straight lines and sharp corners, different parts of the figure will require different strategies, and so adapting the visuomotor control (Julius & Adi-Japha, 2016; Miall et al., 2021). If a participant finds a corner specifically challenging, that will show an increase in overall time performance and, most likely, errors. However, that would be a measure for the whole trial. More fine-grained data can clearly be extracted from the drawings. Detailed performance variations within different regions of the star, as well as more subtle improvements across multiple trials, may be captured. Additionally, traditional MTT measures are restricted to comparing drawings carried out with identical materials, shapes, and test setups.

We propose a quantification approach that yields information beyond the traditional error counts and time spent. It will provide added performance details and enable analyses using varying test material and data sources. Importantly, we propose an approach to facilitate detailed analyses across time points, participants, age ranges, clinical samples, and figure shapes. It will help identify region-specific difficulties as well as constitute a way to compare and measure drawing similarities. For example, this approach may enable the identification of the more cognitively demanding parts of the drawing for individuals or groups, measure how they adapt their traces, explore what those parts have in common, and track how they learn and improve in those specific areas over trials. In essence, this approach aims to enhance the MTT analysis, providing a detailed and spatially resolved assessment of the task's performance. To facilitate its use and implementation, the analysis code is openly available.

Age is a significant factor affecting MTT performance. In age-varying adult populations spanning young, middle-aged, and older adults, age is usually associated with a decline in cognitive or motor performance, as observed cross-sectionally and longitudinally (Kennedy & Raz, 2005; Rodrigue et al., 2005). Both speed and accuracy are known to show negative age relationships and decline with age in the adult lifespan (Fozard et al., 1994), so age is expected to relate positively to time spent and errors committed on the MTT. This is also in line with previous studies of the MTT comparing young and older adults (Brosseau et al., 2007). In order to show the validity of our proposal, we have therefore studied the effect of age on the MTT.

## Methods

### Participants

A total of 210 participants were included in this study. The sample was drawn from the project Set-to-Change, a twin-study project at the Center for Lifespan Changes in Brain and Cognition (LCBC), University of Oslo. The participants were healthy adults aged 16 to 79 years old (36 ± 16 corresponding to the mean and standard deviation). The sample consisted of 145 females and 65 males. Zygosity information was not used in the present work but note that 131 participants were monozygotic (MZ) and 79 dizygotic (DZ), corresponding to 60 MZ and 38 DZ twin pairs and 11 MZ and 3 DZ singles. All the participants who completed their first MTT trial were included.

### Procedure

**Fig. 1:**
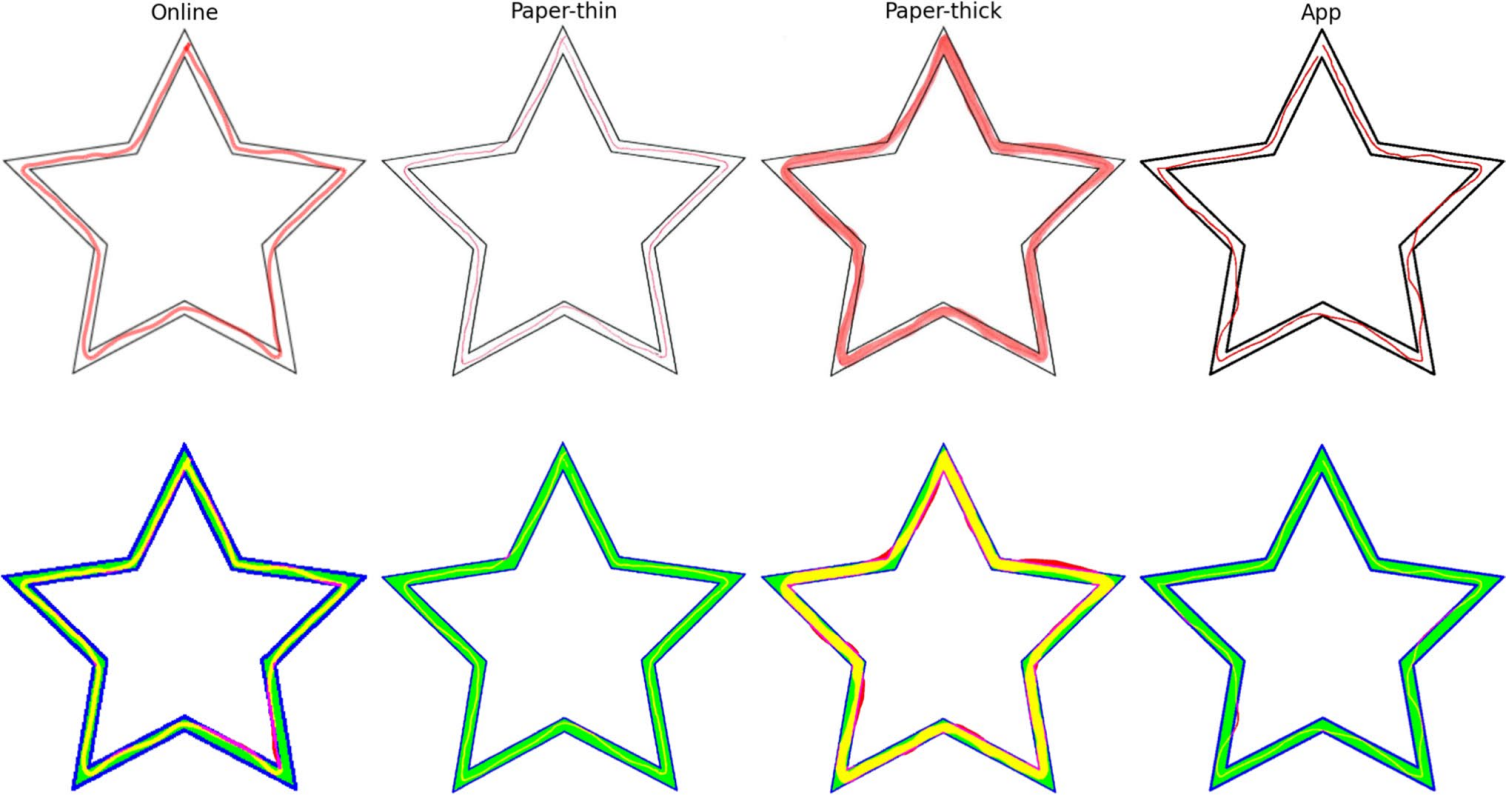
Emulated stars used for clarification of analysis. *Note. Upper row*: emulated stars obtained from the online, paper, and app versions. Paper-thick corresponds to the same star as paper-thin, but highlighted with a marker. *Lower row*: the same stars transformed into the RGB convention. Here, the path, inner star, and border are stored in the red, green, and blue channels, respectively

An image of a five-pointed star with double contour and 18.7 cm width (see examples in Fig. 1) is placed under a box with dimensions 34 cm (length) x 20 cm (width) x 24 cm (height) with a mirror located perpendicular to and in front of the box. The distance between the mirror and the box is 14 cm, and its dimensions are 38 cm (length) x 22 cm (height). The participant is required to introduce their hand into the box and draw, as accurately as possible, within the star’s two borders. With both their hand and the star image covered by the box, the participant relies exclusively on the mirrored reflection of the star to complete the task.

### Materials

Due to practical difficulties and changes over a long project period, three different versions of the MTT material setup have been used in this study. The first version is an online task made free to use by the Project Neuron at the University of Illinois (Project NEURON, n.d.)^1^. Participants using this version performed the task on a tablet computer.

The second version of the task was carried out as a pen-and-paper task. The star used in the online version was recreated, printed out on a white A4 paper sheet, and later digitized for the analysis.

The third version was based on an in-house application and installed on tablet computers. The shape and size of the star were kept identical to the previous ones. In this version, the coordinates of the drawing are saved every 10 ms. To compare results between the versions, only the final image was retrieved from the app, together with the total time spent.

### Image harmonization

For all the images to be comparable and the proposed analysis to be applied with the same conditions, a harmonization of the image is set in an RGB format: the drawn path is stored in the red channel, the inner part of the star would be the green one, and the border is the blue one. The application output already follows these instructions, but those obtained from the web page and the digitized paper version must be transformed. The algorithm created for this purpose has been uploaded to a public repository (Garrido, 2024)^2^. This release includes the conversion of the paper and online stars to the harmonized format, the code for the analysis, and some examples.

### Emulated drawings

In addition to the stars drawn by the participants, some emulated examples have also been created for a better explanation of the analysis pipeline. These stars are shown in Fig. 1 together with their harmonized format. They correspond to well-performed stars drawn on online, paper, and app versions, respectively. For the paper version, a first star was drawn with a pen (paper-thin) and the trace was later highlighted (paper-thick).

## The need for a new approach

**Fig. 2:**
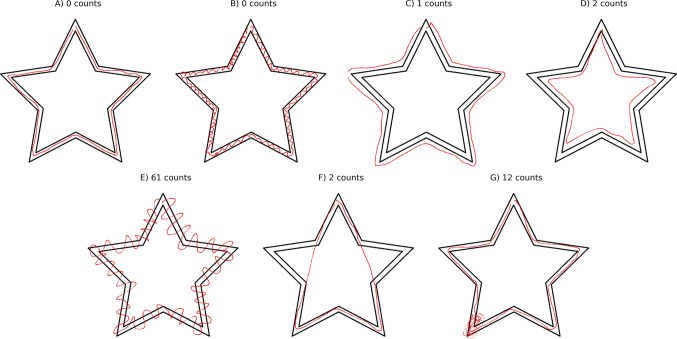
Selection of a well-performed star and six anomalous possibilities on an MTT trial. *Note*. The seven stars were emulated using the app version, each of them showing a particular behavior. **A)** Well-performed star. **B)** Star with oscillations without counts outside the border. **C)** Drawing completely out of the star towards the outside, while stable. **D)** Drawing completely out of the star towards the inside and stable. **E)** Oscillatory drawing along the star with high amplitude and errors both towards the inside and outside. **F)** Drawing with two clear shortcuts. **G)** Well-performed star with a critical error in one of the corners

Another set of seven different stars was created in the tablet application to highlight the relevance of the new proposal over the traditional approach (Fig. 2). This set is a collection of a well-performed star and six anomalous drawings having, by order, (0, 0, 1, 2, 61, 2, 12) counts outside of the main star. These anomalous stars have been created to emphasize and exaggerate the most common errors or difficulties that real participants may have while performing the star. By simply focusing on the number of counts outside, we are losing fine information on the actual performance for these drawings. For instance, star B, with 0 counts outside, reflects an oscillatory behavior that is not accounted for, and its evaluation would be on the same side as star A. According to the goals of the task (to draw inside of the borders and to do it as fast as possible), the assignment is correct, although a suspicious pattern can be observed. Stars C, D, and F, just focusing on the number of times drawn outside, would also correspond to well-performed stars. When comparing star G with A, we may also think that the star was well-done until a certain part, corresponding to a particular region of the star. If this pattern is repeated through different trials or if it is characteristic of a group of people, these are questions that cannot be answered in the traditional way of evaluating the task. Our proposal goes beyond the determination of the number of errors and will provide regional information on the task that can be compared across trials, participants, or even different types of stars.

## Proposed analysis

The new analysis method proposed in this work is based on a polar coordinate system approach. By transforming the data into an angle- and distance-based reference, we can follow the natural movement of the drawing. This transformation allows us to describe the star by means of an analytical function, measure actual distances towards the expected values, and harmonize the measurements. We introduce two alternative variables to measure the task performance: the residuals and the density. Residuals are a variable that accounts for the accuracy of the drawing, measuring, for each pixel, the distance from the ideal trajectory inside the borders of the star. Density contains the information on how many traces a person would need to continue the drawing per unit of angle or per specific region of the reference image. By counting the number of drawn pixels and comparing that with the expected drawing, this variable accounts for the confidence and stability of the performance.

### Polar coordinates, variable description, and generalized star shapes

**Fig. 3:**
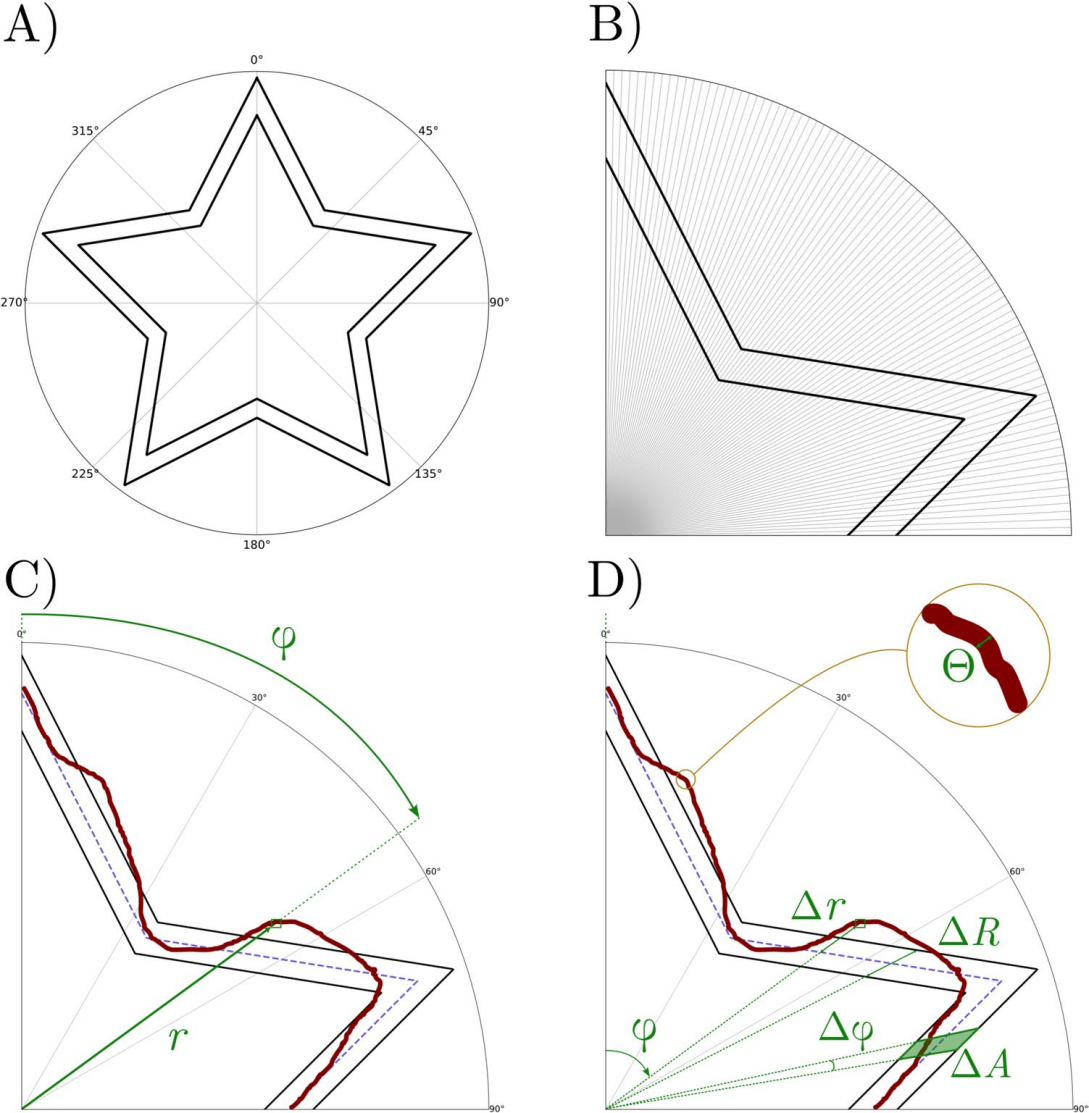
Star figure and drawing in polar coordinates. *Note.* **A)** The MTT star in the polar coordinates representation. **B)** Division of one quarter of the star by 1° slices. The change in the area covered by one slice in the outer and inner corners can be qualitatively appreciated in this image. **C)** Polar coordinates $\left[r,\varphi \right]$(distance to the center and angle from starting point) used to describe the path (in *red*) and the star (the borders in *black*, and the mean star in *dashed blue*). **D)** New variables used to describe the task performance: $\Delta r$-distance between the path and the mean star; $\Delta R$-width of the star for a specific angle; $\Delta \varphi$-angular displacement; $\Delta A$- area covered by an angular displacement; *Θ*- thickness of the drawing path, in pixels

**Fig. 4:**
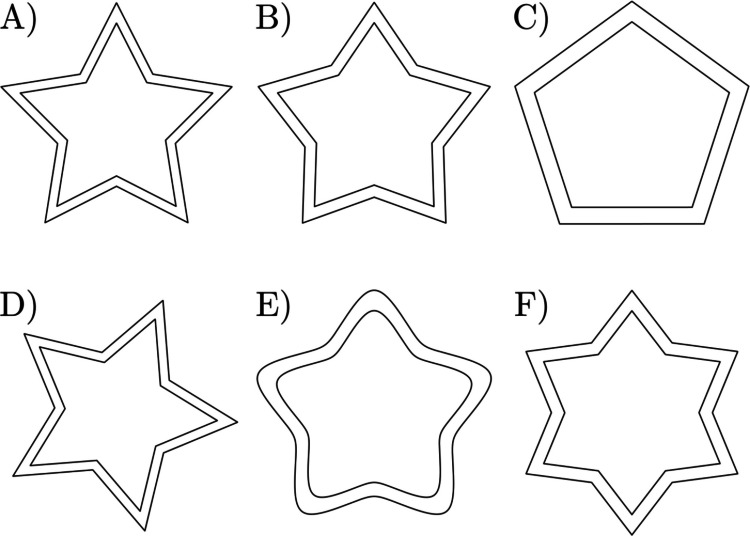
Selection of 6 different stars that can be described by Eq. (1). *Note.* This figure contains a selection of a set of possible shapes that can be analyzed by our method. Any combination or addition to any of them can also be implemented. **A)** Original star used in this work. **B)** Original star with side-bending modification. **C)** Original star with side-bending modification reproducing a polygon. **D)** Original star with rotation. **E)** Original star with peak smoothness modification. **F)** Original star with an extra peak

The usual [*i,j*] pixel coordinates of a digital image are transformed into polar coordinates [*r*, φ] where *r* is the distance from the center of the star towards a particular pixel and φ the angle from the starting drawing point (top of the star) and measured clockwise (see Fig. 3A,C). This change of coordinates enables the description of the shape of the star as a function, giving a unique distance value for each angle. A first attempt was made through the function known as the *superformula*, proposed by Johan Gielis (Gielis, 2003). However, it could not properly define the corners of the star as they are too smooth. We tried another proposal found in a non-peer-reviewed source, with outstanding results not only in describing the shape of the stars we tried to analyze, but also many other kinds of star shapes (Fig. 4 and Figures S1–4) (Sokol, n.d.)^3^. The equation, with some small modifications, relates the distance of any point on the border of the shape to the angle described around the center of the figure (and measured in radians) as:

1 $$r\left(\varphi \right)={r}_{0}\frac{\mathrm{cos}\left(\frac{2{\mathrm{sin}}^{-1}\left(k\right)+\pi m}{2n}\right)}{\mathrm{cos}\left(\frac{2{\mathrm{sin}}^{-1}\left(k\mathrm{cos}\left(n\left[\varphi +\upomega \right]\right)\right)+\pi m}{2n}\right)}$$

where ${r}_{0}$ represents the mean distance to the center of the star, $\omega$ is a phase shift to account for a rotation (when scanned, for instance), $k$ is a parameter that controls the roundness of the peaks, $m$ is the side bending or depth, and $n$ is the number of vertices.

In addition to the new pair of coordinates, new variables can be introduced and used to describe task performance (Fig. 3D). Based on the new coordinate system, we can define, for each pixel, the distance (*r*) towards the center of the star and the angle (φ) from the starting point. Based on the function that describes the star, we can determine, for that angle, the mean value inside its borders, which we call the ideal star. We can now calculate the difference between the actual path and the ideal one. Let Δ*r* be this difference. At the same time, we can measure, for that angle, the distance between the borders of the star, i.e., the width for a specific angle. This distance varies along the shape as the star width is smallest in the parts closest to the center. Let Δ*R* be that distance. For a given pair of angles covering an angular displacement Δφ, we can define the area covered inside the star by that movement as Δ*A*. Finally, the last variable to be introduced is the path thickness,Θ. This variable is of special interest when comparing tasks with different pencils, pens, or markers, and it is measured in the proposed algorithm.

### Ideal and expected stars

As briefly described in the previous section, we introduce the ideal star as the mean value inside the borders. According to Eq. (1), if ${r}_{ext}$ and ${r}_{int}$ correspond to the mean radii of the external and internal borders of the star, the ideal star would be described as:

2 $$r\left(\varphi \right)=\frac{{r}_{ext}+{r}_{int}}{2}\frac{\mathrm{cos}\left(\frac{2{\mathrm{sin}}^{-1}\left(k\right)+\pi m}{2n}\right)}{\mathrm{cos}\left(\frac{2{\mathrm{sin}}^{-1}\left(k\mathrm{cos}\left(n\left[\varphi +\upomega \right]\right)\right)+\pi m}{2n}\right)}=\frac{{r}_{ext}+{r}_{int}}{2}f\left(k, m,n,\upomega ,\varphi \right)$$

where $f\left(k, m,n,\upomega ,\varphi \right)$ is the function that describes the geometrical shape of a given star except for the scaling factor.

**Fig. 5:**
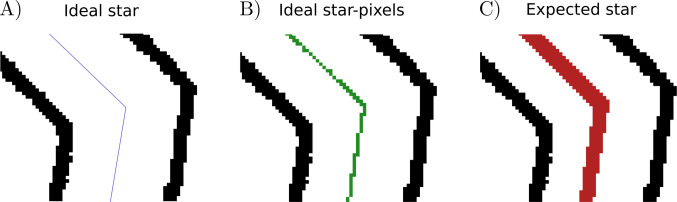
Ideal and expected star comparison. *Note.* Graphical comparison of ideal and expected stars used in our method. To highlight the effect of the pixels, a region around the corner of a digitized image has been selected. The borders of the star are kept with their original pixel shape and represented in *black*. **A)** Ideal star determined as the mean value between the borders and calculated using Eq. (2). The ideal star is represented as a line with no width. **B)** Ideal star in the corresponding pixel resolution. The ideal star is represented in each of the pixels the line goes through. **C)** Expected star determined as the ideal star in the pixel representation and expanded up to the original thickness of the drawing

Equation (2) can be used to recreate the ideal star in polar coordinates (Fig. 5A), needed for the residuals’ calculation. If we want to make use of it in the real image, it must be transformed back to pixel coordinates $\left[i,j\right]$ by reversing the polar transformation and collecting only the integer values (Fig. 5B). From this representation in pixels, the expected star is defined as the dilated ideal star up to the measured thickness of the drawing $\Theta$ (Fig. 5C). The expected star aims to reproduce the ideal star if it were done with the same drawing utensil as the original task.

### Residuals

The $\Delta r$ is already a valid magnitude to measure how far the drawn path deviates from the ideal star. It can also be used to compare different drawings using the same kind of star. However, to compare similar stars with uneven sizes or to compare different star shapes’ results, $\Delta r$ must be harmonized. The harmonization is done simply by the ratio of this distance difference and the distance between borders, $\Delta R.$ We define this new magnitude as the residuals, $\Delta {r}{\prime}$ (Eq. 3). Using this definition, the residuals are considered positive towards the outside and negative towards the center of the star, and are 0 if the pixel is located on the path of the ideal star. This measure additionally reduces the effects related to the shape of the star in the analysis. It is common to draw farther from the mean star when the borders are wider, for example. Using this representation, the borders of any of the possible star shapes would be a circumference.

3 $$\Delta {r}{\prime}\left(\varphi \right)=\frac{\Delta r\left(\varphi \right)}{\Delta R\left(\varphi \right)}$$

### Density

Density is a variable defined to show how many pixels are drawn in a certain area of the star. For this reason, it is a regional variable, and therefore it must be measured between pairs of angles rather than per pixel. We define Δφ as the angular resolution corresponding to the size of the regions we want to examine. In Fig. 3B, the 1° resolution is shown as an example. For a given interval, we can count the number of pixels drawn in it, independently of whether they are inside the star or not (the precision or accuracy is measured by the residuals). The first proposal, which was discarded, was to standardize the measurements across different stars by dividing the number of pixels by the area of the star in that interval and the thickness of the path (Eq. 4).

4 $${\rho }_{old}\left(\varphi ,\Delta \varphi \right)=\frac{\#pixels(\varphi ,\Delta \varphi )}{\Delta A(\varphi ,\Delta \varphi )\Theta }$$

However, as previously addressed, the width of the star changes with the angle, and so does the area. This condition is especially problematic around the corners of the star, where the density increases as the area contains two parts of the path, or is too narrow, which would artificially overestimate the density. To avoid this effect, the number of pixels is harmonized by the number of pixels for that region in the expected star, $E\left[\#pixels\right](\varphi ,\Delta \varphi ,\Theta )$. Following this last step, using a thinner or thicker pen will have no influence on this variable. We denote this density by $\rho$, and it is defined as follows:

5 $$\rho \left(\varphi ,\Delta \varphi \right)=\frac{\#pixels(\varphi ,\Delta \varphi )}{E\left[\#pixels\right](\varphi ,\Delta \varphi ,\Theta ) }$$

### Time series-like approach and data clustering

A time series data structure corresponds, briefly, to a variable that is measured at regular intervals, but not restricted to time. Due to the lack of independence of each measurement of the variable, most statistical tools cannot be directly applied to its analysis and therefore, specific methods have been developed to solve the different questions that emerge from them. One of these questions could be measuring similarity between time series, which can later help in clustering of drawings. We propose the use of the time series data approach to analyze and compare the data obtained from every drawing, where our independent variable would be the angle around the center of the star.

Density is already measured per unit of angle. Residuals can also be summarized in the same way if we consider, for example, the mean residuals, the mean absolute residuals, or the mean squared residuals for the same interval. When the newly introduced variables are measured per unit of angle, per region, or per angle interval, we obtain a multivariate time series-like data. By means of this approach, we can focus our analysis on specific parts of the star. Additionally, the time series approach facilitates the comparison of drawings and allows for the use of similarity metrics that account for the non-independence of the values. Time series clustering techniques can help identify groups of patterns repeated by the same person or a group of people. Grouping similar drawings facilitates the interpretation of the different patterns that may appear in our data sample. As a final remark, the time series similarity metrics also provide a framework for quantifying the similarity between a group of drawings. Beyond their use for clustering, these metrics can also be used to create classification models.

### Data analysis

All data preprocessing, curation, and analysis have been performed in Python, with scripts publicly available and converted into a Python package named *ursamirror* (Garrido, 2024). Both these scripts and the analysis done for this work are based on several Python packages: *NumPy*, *SciPy*, *statsmodels*, and *pandas* for data manipulation and analysis (Harris et al., 2020; McKinney, 2010; Seabold & Perktold, 2010; The pandas development team, 2024; Virtanen et al., 2020); *Matplotlib* and *seaborn* for data representation (Hunter, 2007; Waskom, 2021); *tslearn* for time series data analysis (Tavenard et al., 2020); *scikit-image* for image-specific analysis (Walt et al., 2014).

Linear mixed effects models (LMMs) with random intercepts were used to study the associations between variables while accounting for the non-independence of the twin dataset (Carlin et al., 2005), with a significance level of 0.05 and 95% confidence intervals. The regression coefficients of age for *Z*-scored errors, time, residuals, and density were compared using a bootstrap approach, resampling twin pairs with replacement 1000 times, yielding 1000 new datasets with the same number of twin pairs as the original dataset. On every resampling, a new LMM is fitted, and the corresponding regression coefficients of age are collected and compared. The *p* value of the comparison between all coefficients was estimated based on the proportion of times one coefficient is larger than the others. When comparing multiple tests, the *p* values were corrected using the false discovery rate (FDR) approach and the Benjamini–Yekutieli method (Benjamini & Yekutieli, 2001).

Multivariate time series clustering was performed using the *k*-means algorithm, applying three different similarity metrics: dynamic time warping (DTW), constrained dynamic time warping (c-DTW), and Euclidean distance (ED). The first two metrics are time series-specific, which allow time (angle in this work) shifts in the data. Based on each similarity metric, representative time series-like data from every set of stars were obtained by means of the barycentric approach, using the appropriate metric. The barycentric technique aims to determine the centroid of a group of time series data, providing an average representation by minimizing the square distance between all the series in the dataset (Petitjean et al., 2011; Sakoe & Chiba, 1978; Schultz & Jain, 2018; Zhang et al., 2017). When using the ED, the barycenter corresponds to the mean value for each measured angle. However, when applying time series-specific metrics, the barycenter can also account for displacement or shifts, with the metric to be minimized being the DTW or c-DTW in our examples. The association between cluster demographics and sex was evaluated using the χ2 test, and the association with age was assessed using the Kruskal–Wallis test.

## Results

### Harmonization of the magnitudes

**Fig. 6:**
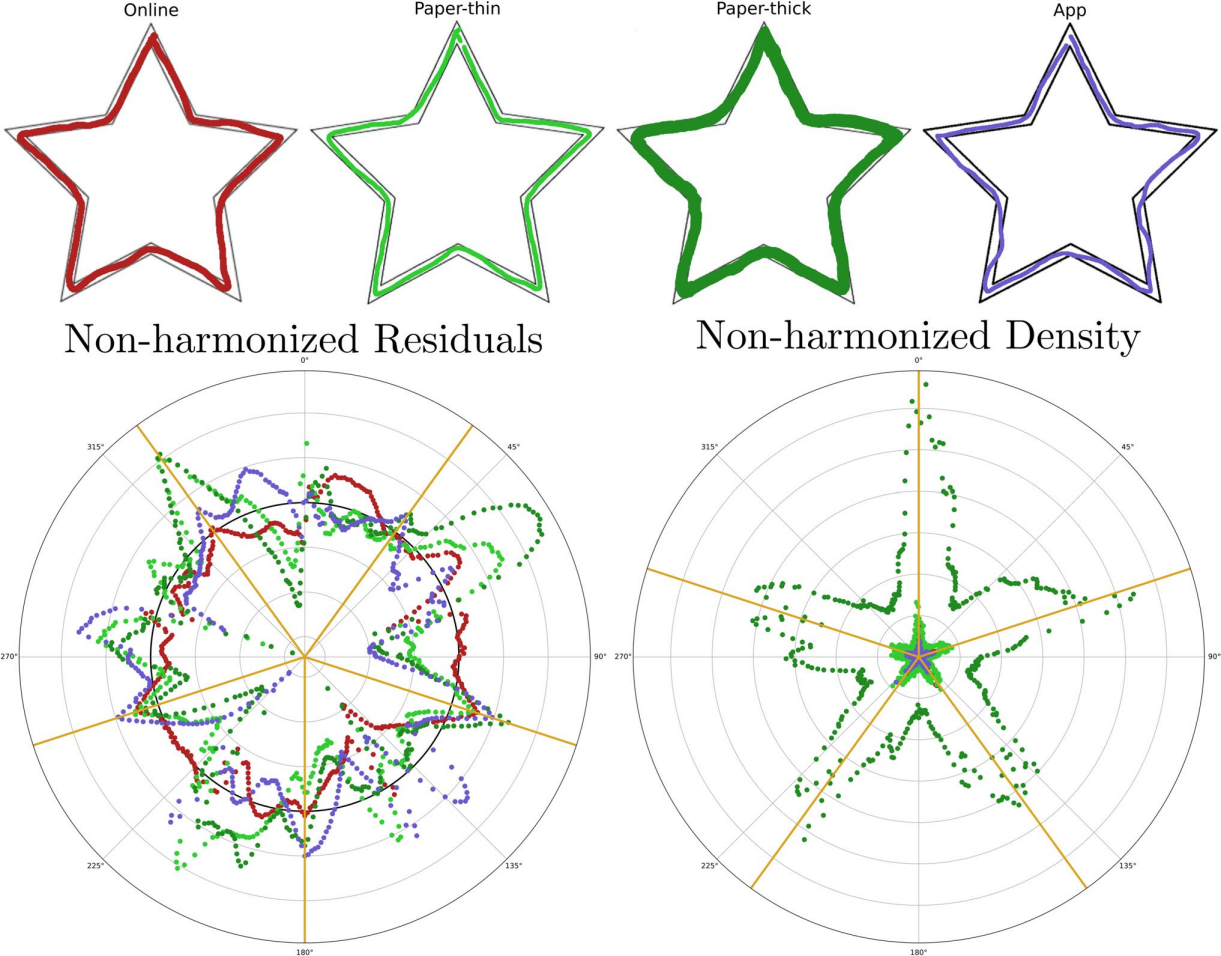
Non-harmonized results for the emulated stars from different MTT versions. *Note. Upper row*: stars proposed in Fig. 1. The stars represent each of the set-ups used in this work: online, paper, and app. Paper-thick corresponds to the same star as Paper-thin but highlighted with a marker. The drawings are shown with the color code used in their representation (*red*-Online, *light green*-Paper-thin, *dark green*-Paper-thick, *blue*-App). *Lower-left*: Non-harmonized residuals in the polar coordinates’ representation. The non-harmonized residuals are calculated as the difference between the ideal star and the actual drawn path, with no correction for the star width ($\Delta R$). The *black circle* corresponds to the 0-value residuals. The *yellow lines* indicate the five directions corresponding to the inner corners of the star. *Lower-right*: Non-harmonized density in the polar coordinates’ representation. This non-harmonized version only counts the number of pixels for every angular displacement $\Delta \varphi$. The *yellow lines* indicate the five directions corresponding to the outer corners of the star. The values of the paper-thick star mask the other values, due to the non-harmonized data

After defining the new variables (residuals and density), we examined the effect of harmonization in the stars on the different versions of the MTT. The four stars presented in Fig. 1 (online, paper thin, paper thick, app) would be considered well-performed by means of the traditional method of evaluation as they have at most one or two counts outside the borders. Now, we aim to compare them by means of the new set of variables. The results using the non-harmonized variables, without correcting for star width on the residuals and the expected number of counts for the density, are shown in Fig. 6. The angles of symmetry in the star create artifacts in both the residuals and the density. The inner corners increase the value of the residuals, while the outer corners show increased density values as they contain more traces of the path. Additionally, density is especially sensitive to the effect of the thickness of the drawing.

**Fig. 7:**
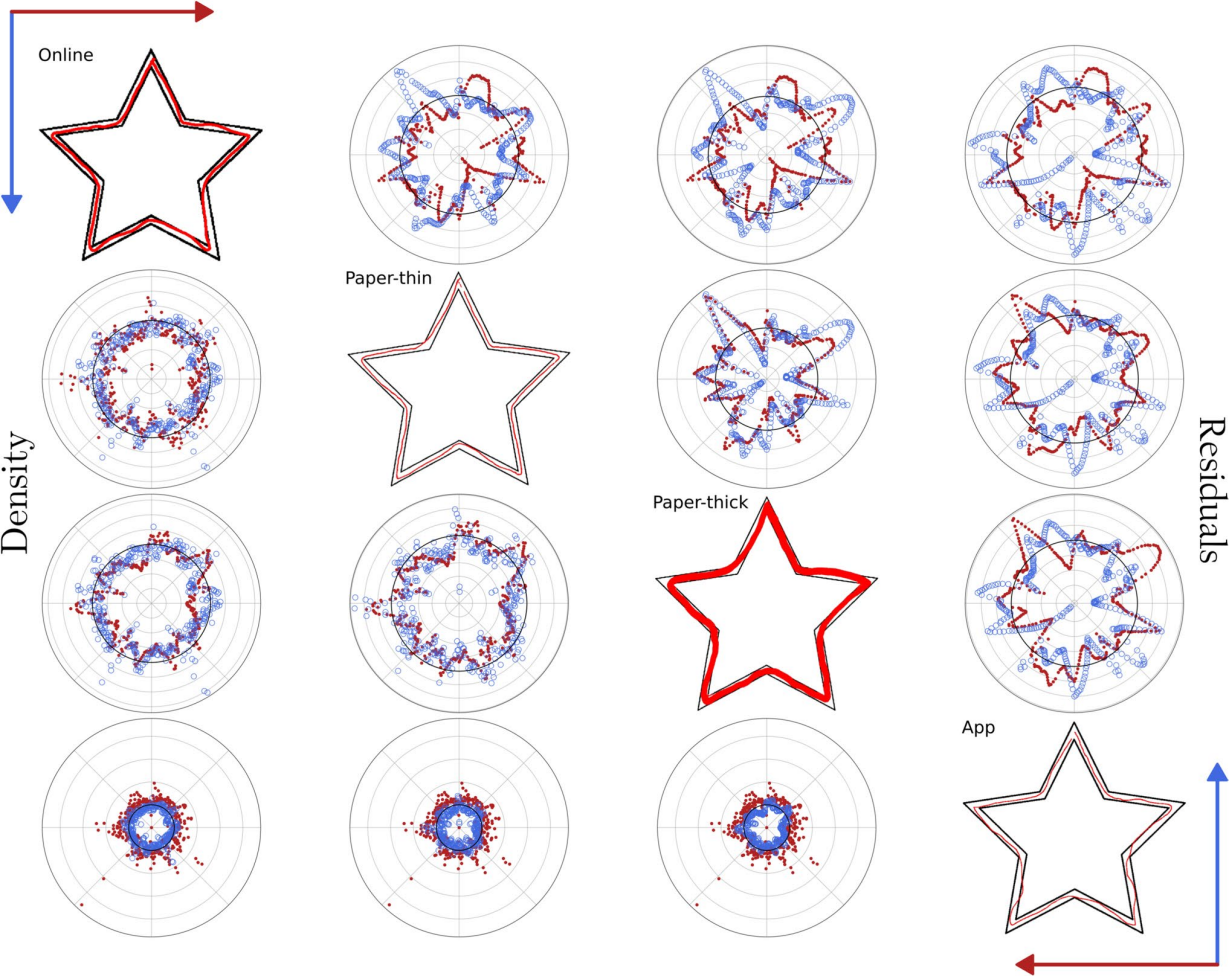
Comparison of the four emulated stars from different MTT versions using the harmonized variables*. Note.* Compact representation and comparison of both residuals and density obtained for the drawings representing the three versions of the MTT presented in the paper: online, paper, and app. Paper-thick corresponds to the same star as paper-thin but highlighted with a marker. The *upper triangle* of the matrix plot corresponds to the residuals, representing the difference between the ideal star and the actual drawn path, corrected by the star width (ΔR). The *lower triangle* gathers the density comparison, which accounts for the number of drawn pixels per angle and is referred to the expected drawing. The values for the star in the diagonal are plotted in *red* in its own row and *blue* in its own column.

Once the harmonization is applied to the variables, they become easily comparable across the four versions, all of them falling within the same range of values, as shown in Fig. 7, Figures S5 and S6, and summarized in Table S1. When comparing both paper versions, it is noteworthy that *paper-thick* is exactly the same drawing as *paper-thin* but highlighted. The harmonization of the density reveals the same pattern and almost the same values when comparing them, as does the harmonization of the residuals. The star obtained from the app version is the only one that seems to be outside of the normal range in both density and residuals, as it has an error just after the bottom-left corner. The polar coordinates representation allows us to more accurately identify the region where the error appears.

### Comparison of anomalous star drawings

**Fig. 8:**
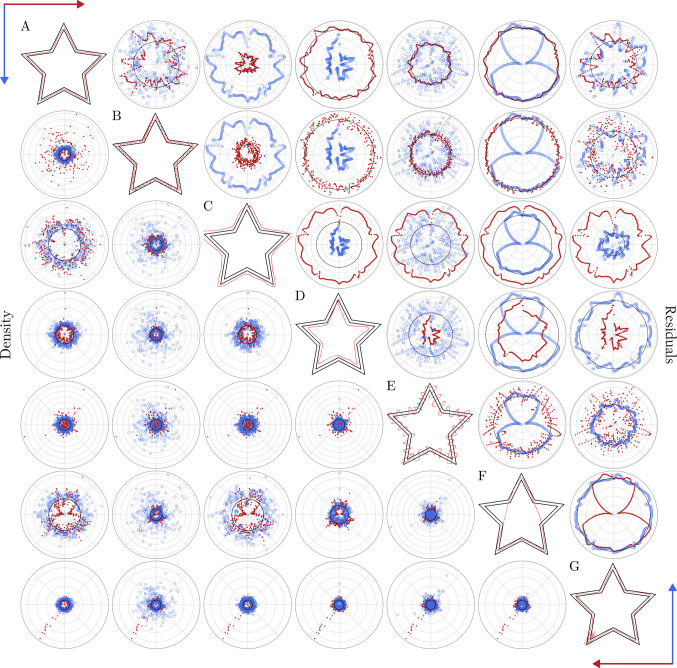
Comparison of the six anomalous emulated stars from the app version using the harmonized variables. *Note.* Compact representation and comparison of both residuals and density obtained for the well-performed star (**A**) and six anomalous drawings (**B**–**G**). Stars **B** and **E** represent two oscillatory patterns with different amplitudes. Stars **C** and **D** correspond to two cases where the drawing is completely out of the star but preserves its shape. Star **F** represents a drawing with two clear shortcuts. Star **G** represents a well-performed star with a located error in one of the corners. The *upper triangle* of the matrix plot corresponds to the residuals, representing the difference between the ideal star and the actual drawn path, corrected by the star width (ΔR). The *lower triangle* gathers the density comparison, which accounts for the number of drawn pixels per angle and refers to the expected drawing. The values for the star in the diagonal are plotted in *red* in its own row and *blue* in its own column.

The introduction of the new variables opens a new way of understanding and evaluating the set of anomalous drawings presented before. We can now compare all of them, on the same scale and by angle or region (Fig. 8), analyze their value distributions (Fig. S7), or summarize them in single values (Fig. S8 and Table S2). Using star A as the reference well-performed star, star B has a similar residuals distribution, but the density is significantly higher across almost all the angles, as a result of the oscillatory behavior. Stars C and D are comparable to star A in terms of the density, but their residuals are highly positive or negative. Star E has both high density and high residual variance, as a reflection of its large amplitude oscillation. Star F shows the effect of a shortcut in both residuals and density. Finally, star G is a good example of the patterns that may show problems in specific regions. For this star, both the residuals and the density behave similarly to star A, until the region around 216°, where the density drastically increases.

### Summarized residuals and density as alternative measurements

**Fig. 9:**
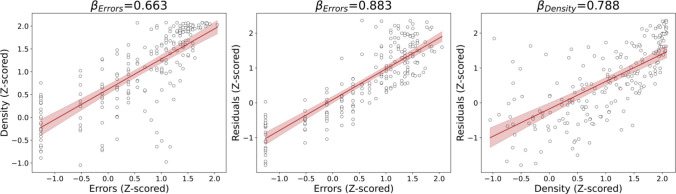
*Z*-scored comparison between summarized density, residuals, and errors for the 210 participants. *Note.* Comparison of the *Z*-scored Errors (the traditional measurement) the summarized Density (expressed as the mean density value for each drawing) and Residuals (determined as the sum of the squared residuals for each drawing). For each comparison, the corresponding correlation coefficient from the LMM is shown at the top of each plot, all of them corresponding to a *p* value of < 0.05. The linear fit is represented with a solid red line with a shaded region representing the 95% confidence interval around it.

Residuals and density are introduced to replace the traditional evaluation based on counting the number of times a person draws outside the border of the star (the Errors). The main advantage of these two variables is the fact that they can be measured by angle or region, while also being continuous numeric variables. However, if we want to compare them directly with the number of errors, they must be summarized as a single value for every star. We decided to use the mean density and the sum of the squared residuals. These two summarized values and the errors are normalized by a Box-Cox transformation and standardized (*Z*-scored) for their comparison (see Fig. 9). The summarized residuals capture most of the information from the number of errors, and they are complemented by the summarized density, which is also linearly associated with the errors. Additionally, density and residuals are linearly correlated as summarized variables. The sum of the squared residuals has been chosen over the mean squared residuals, the mean residuals, or the sum of the residuals. Working with the residuals as a summarized variable is less informative as the positive and negative values can compensate each other, resulting in summarized values closer to 0. By using the squared values, the sign is no longer relevant. The difference between using the mean or the sum of the residuals is just a scaling factor.

**Fig. 10:**
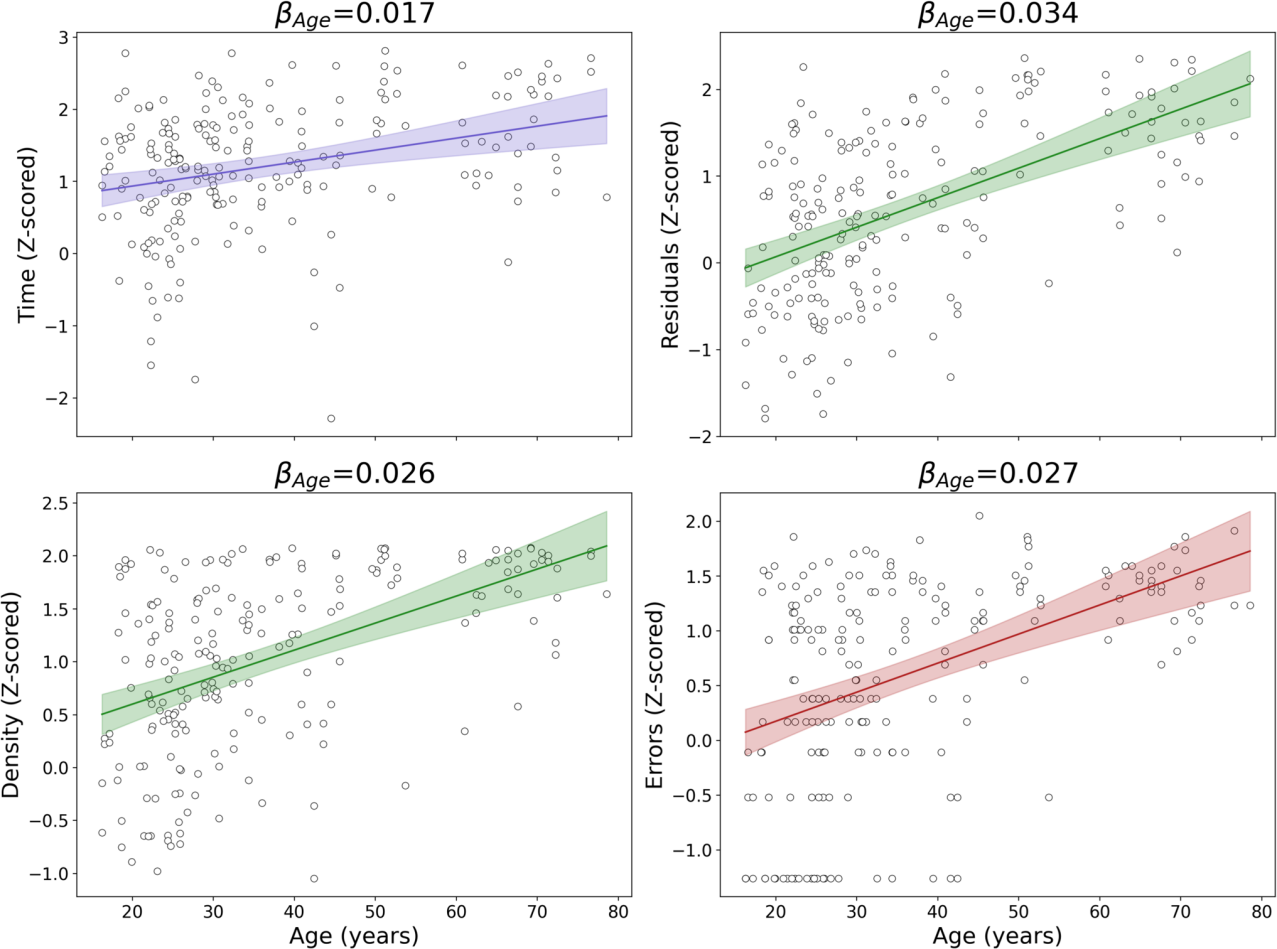
Comparison of *Z*-scored time, summarized residuals and density, and errors and its evolution with age. *Note.* Comparison of the *Z*-scored Time to complete the task, Errors (the traditional measurement), summarized Density (expressed as the mean density value for each drawing) and Residuals (determined as the sum of the squared residuals for each drawing) and their correlation with age. For each comparison, the corresponding correlation coefficient from the LMM, all of them corresponding to a *p* value < 0.05. The linear fit is represented with a solid line with a shaded region representing the 95% confidence interval around it: blue for Time (the common variable that can remain from the old approach to our proposal), green for Residuals and Density (the new variables), and red for the Errors (the variable to be replaced)

As a proof of concept, we tested these summarized variables, the number of errors, and the time used to complete the drawing, with age as a variable of interest (Fig. 10). Time was the least related to age in this comparison, density and errors showed similar relationships, and residuals were slightly more strongly age-related, meaning they can be more sensitive to age. A more rigorous comparison in our sample using the bootstrap method states that the correlation of Residuals with age was statistically significantly larger than the other coefficients, while the correlation coefficient of Time with age is significantly smaller (see Fig. S9). It is worth mentioning that both residuals and density are measured together in an independent way, and so they can complement each other.

### Residuals and density as a function of the angle. Time series analysis and sensitive regions with age

**Fig. 11:**
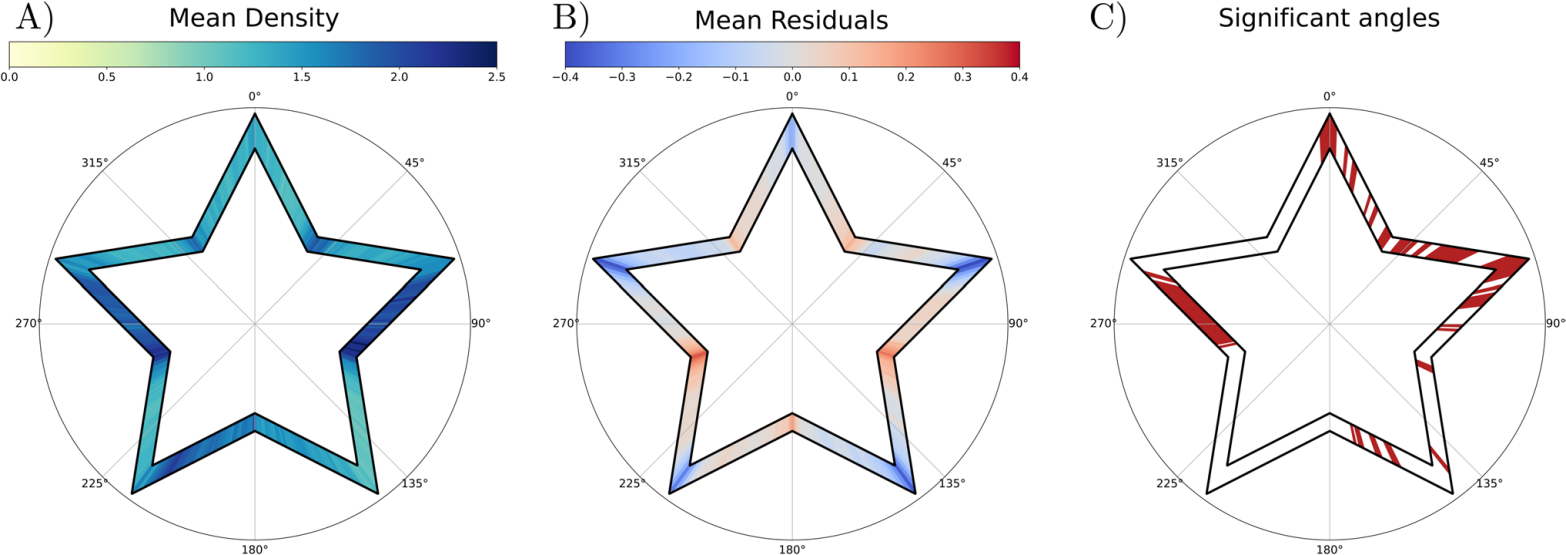
Polar coordinates representation of the mean density, mean residuals, and statistically significant regions with 1° angle resolution. *Note.* Descriptive statistics of the MTT performance in our sample. The use of our approach allows us to perform the analysis per angle. **A)** Mean Density values of our sample per angle. Colormap from yellow to dark blue (0 to 2.5). *Darker regions* show those parts of the star where our participants have, on average, drawn less straight lines. **B)** Mean Residuals values of our sample per angle. *Blue to red* (– 0.5, inwards, to 0.5, outwards), with 0 corresponding to *white*. The *darker values* in the corners show those parts of the drawing where people tend to, on average, take shortcuts in the path. **C)** Significant angles: in *red*, those angles where both the density and the residuals have a statistically significant linear correlation with age at the same time. After appropriate correction of the *p* value, the highlighted angles correspond to those < 0.05.

Beyond the comparison with the number of errors and summarized residuals and density, the main advantage of this new approach is the analysis per angle or region. As an example of this application, both the mean density and residuals were analyzed per angle for the first MTT trial out of the 210 participants (Fig. 11). We selected just the first trial to avoid practice effects. As expected, not all angles have equal values. There are regions that, on average, are more difficult to solve, especially around (but not restricted to) the corners.

For the density values, both the symmetric inner corners around the starting point (36° and 324°) showed increased density, meaning that the average person would need more traces on that part to continue the drawing. The side just after the outer corner at 72° and just before 288° also showed higher density. However, this apparent symmetry is broken around 180°, as the density is increased just after that corner, but not before. These emphasized regions would correspond to those parts of the star where, on average, people are less confident in their performance.

For the residuals, the same kind of symmetry and asymmetry that was reflected in the density was also observed. The details that this variable provides on top of the density concern the direction of the errors and how far they are from the ideal star. On average, people tend to draw more towards the center of the star in the outer corners (negative values) and more towards the outside in the inner parts (positive residuals). The inner corners around 108° and 252° seem to be less accurate than the other corners, as the residuals are higher in these areas. At 180° an asymmetry can be observed, as previously described for density. The sign of the residuals changes preceding and following the corner.

The detailed regional analysis can be extended beyond a mere description of the population. We can identify the angles or regions where both the density and the residuals have a statistically significant linear correlation with age (FDR-corrected *p* value < 0.05 using Pearson’s correlation test and Benjamini–Yekutieli method). The results are shown in the last plot of Fig. 11. The beginning of the drawing and the first inner and outer corner had the strongest correlation with age, as well as the side around 270°.

### Star clustering by time series approach

**Fig. 12:**
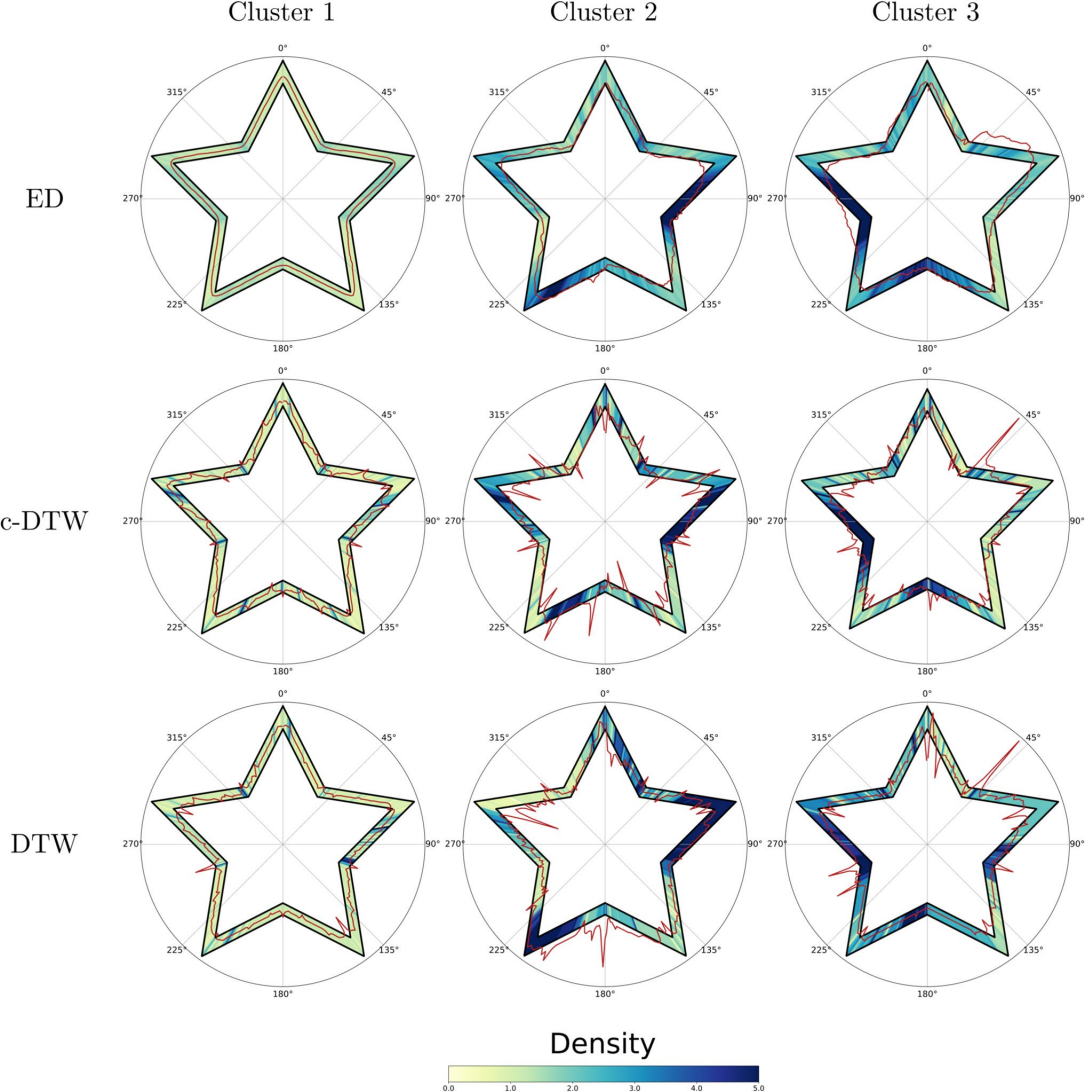
Matrix plot of the barycentric representative star for each cluster and metric approach. *Note.* Representative stars for each cluster and metric, obtained by the barycentric method. *ED* Euclidean distance, *DTW* dynamic time warping, *c-DTW* constrained dynamic time warping. Both the residuals and density are represented together for a more compact visualization. The density is shown in a color scale (from 0 to +5). Residuals are drawn with a *red line*. Clusters 1, 2, and 3 correspond to the clusters grouping the best, worst, and mid-drawn stars, sorted for each metric

The last application shown in this paper corresponds to the unsupervised clustering of the stars. We applied three different approaches to our sample, looking for a classification of the 210 stars into three clusters. After the stars were sorted, the barycenter approach was applied to obtain a representative image of each cluster. For the three clustering techniques, Cluster 1 corresponds to the set that collects the stars that are well performed. Cluster 2 groups the stars that have some errors, although less pronounced than those of Cluster 3, which shows a considerable amount of errors in their performance (Fig. 12).

The results show that any of the methods creates a cluster of well-performed stars (Cluster 1), where the traces are, on average, inside the borders and the density is generally low. The characteristics of the two remaining clusters vary depending on the metric, although they share some common traces. Cluster 2 contains drawings that, on average, tend to show more difficulties between the first outer corner and the second inner one (72° to 108°), and the third inner corner and the outer one (180° to 216°). The drawings in Cluster 3 have larger residuals starting around the first inner corner (36°), and characteristic high densities at the third and fourth inner corners (180° and 252°). An increased value of density reflects an increased number of retraces for those regions, showing low confidence and more difficulties.

The most suitable metric will depend on the research question and the level of precision in the angles. The time series approach enables the use of clustering techniques focused on fine-grained similarities (Euclidean distance) or more on overall performance (DTW), with different levels of detail in between, as approached by the constrained DTW. We analyzed the demographics (sex and age) of each group for every metric, but the clusters are highly unbalanced (see Table S3). Cluster 1 contains more than 80% of the drawings using any of the metrics, as most of the stars are well performed. Statistical tests have been used to identify the existence of an association with age and sex, although these results should be considered as merely indicative due to the high imbalance. No statistical significance was achieved when examining sex independence (all *p* values > 0.05), while age suggests statistical differences among all the groups for any of the metrics (*p* values < 0.001). Cluster 1, for example, is associated with a younger sample compared to the other clusters across the three tested metrics (see Figure S10).

## Discussion

The Mirror Tracing Task has traditionally been evaluated by measuring the number of errors and the time to complete the task. Other metrics have been used to estimate performance, often combining or deriving from these primary measurements. For instance, time off is defined as time spent outside of the pattern instead of the total time (Backhaus & Junghanns, 2006; Gabrieli et al., 1993). Additional metrics, such as the product or the ratio between errors and time, account for the “speed–accuracy trade-off ” (Bornovalova et al., 2008; Dumel et al., 2015; Feldman et al., 2014; Veilleux et al., 2019). A metric used for unfinished tasks is the error-side ratio, determined as the number of errors divided by the number of completed polygon sides (Julius & Adi-Japha, 2016; Vicari et al., 2005). Path length, defined as the total length of the drawn figure or total displacement of the pen (Lemieux et al., 2014; Miall et al., 2021), as well as the number of times a participant changes the direction of the drawing (Ferrel-Chapus et al., 2002; Julius & Adi-Japha, 2016), have also been used. However, these approaches reduce the whole drawing to one single value, lacking precision to identify specific details or specific mistakes. In contrast, our method introduces a new perspective in measuring performance by defining the residuals (the deviation from the ideal path) and the density (how often a specific path is retraced) per angle, enabling a regional analysis while keeping the possibility of using them as a summarized variable.

### Image and shape harmonization

Our goal was to make the method as standard as possible, so it can be extended to any star or regular shape, as well as to various image sources. This standardization facilitates the reanalysis of previously collected data, feasible through a harmonization proposal. We suggest starting by transforming any digitized image of the MTT to a new image of any format, but using the RGB channels to store the path, inner star, and borders of the star, respectively. It will help with the analysis and the organization of the data. Several cases are already supported in the provided Python package (Garrido, 2024), easing the transition from the digitized paper-based or the online setup to this suggested format. This system ensures a consistent format, independently of the source. For instance, different set-ups can be found in the literature. The most common one is paper-based, which can be digitized afterwards, but digitizing tablet- or computer-based tests are becoming more commonly used in the later years (Alrubaye et al., 2024; Balslev et al., 2004; Feldman et al., 2014; Lemieux et al., 2014; Taghian et al., 2025). For most of them, the same RGB transformations are still valid and easy to implement.

Another source of variation in the MTT design is the shape of the Fig. used in the test. Five- or six-pointed stars are commonly employed (Alrubaye et al., 2024; Azhari et al., 2024; Finn et al., 2016; Gabrieli et al., 1993; Lemieux et al., 2014; Vicari et al., 2005), although squares, circles, and other more complex figures have also been used (Gullaud-Toussaint & Vinter, 2003; Julius & Adi-Japha, 2016; Miall et al., 2021; Salowitz et al., 2013; Waldrop et al., 2001). With our approach, the quantitative method of residuals and density can be extended to any star or regular shape, which includes a large spectrum of possibilities in the MTT. As shown in Fig. 4 and Figures S1−4, multiple stars are considered, including variations or combinations of the different tuned features. Even the commonly used five-pointed star has variations, especially in the angles that define the corners, which changes the difficulty of the task. Overall, our method can be extended to any of the set-ups or shapes, facilitating comparisons of tasks in different studies and modalities.

### Summarized variables

The introduction of the new variables as a function of the angle can be summarized as single values and used to describe the drawing. This is the only way to compare them with the traditional measurements, such as errors. For this purpose, we used the mean density and the sum of the squared residuals. Intuitively, the value of the summarized residuals should be in line with the errors, as the higher the number of errors, the further away the drawing is from the ideal star, increasing the residuals. As shown in Fig. 9, residuals are highly correlated with errors, as expected. However, the correlation of density with errors is lower. Both residuals and density refer to the MTT performance, but they capture different features. If we focus on the anomalous stars presented in Figs. 2 and 8, as well as in the summarized metrics in Figures S7 and S8 and Table S2, the oscillatory pattern presented in Star B has residuals values close to 0, as does the number of errors, but exhibits high density. This type of pattern has already been identified in previous works, and it is difficult to identify solely based on errors (Lemieux et al., 2014; Miall et al., 2021; Rouleau, Décary, et al., 2002a, 2002b; Rouleau, Salmon, et al., 2002a, 2002b). However, when including the summarized density, the drawing stands out (Figures S7 and S8).

Residuals and density are two complementary variables that can tell us more about the drawing than just the errors. As stated in this work, there exist several limitations when reducing the whole task to single values instead of looking at specific parts of the star. Star G in Fig. 8 is an example of these limitations, as one single located error does not move the density nor the residuals values out of what is expected for a well-performed star (Figures S7 and S8 and Table S2), although the standard deviation for both increases. The difference becomes more pronounced in the angle analysis (Fig. 8), particularly when utilizing the capabilities of the new approach.

### Age relations of traditional and new MTT metrics

As expected based on previous findings (Brosseau et al., 2007; Fozard et al., 1994; Julius & Adi-Japha, 2016; Kennedy & Raz, 2005; Rodrigue et al., 2005), both time to completion and number of errors correlated significantly with age in the current sample. We have thus used age as a variable of interest also with this new approach and compared age relations of new metrics with those of time and the number of errors. Both the residuals and the density are correlated with age, in agreement with the results using the number of errors, when considered as individual variables. Our analysis indicated that the residuals were more strongly related to age than were other variables, indicating this metric may be of value for targeting and understanding age-related performance differences. When analyzing the data by angle, the new protocol shows the regions that become more difficult with age. Such analyses may reveal interesting patterns, since, as pointed out in previous findings, the variability of motor skill learning may increase with age, and specific performance deficits may associate with specific neurocognitive difficulties (Brosseau et al., 2007).

### Clustering analysis

The unsupervised clustering used in this work should be considered as a possible extra application of the new variables that were not feasible before. Introducing the time series-like approach opens the possibility for using time series-specific analysis, like the metrics used for clustering. Both the use of the Euclidean distance and the DTW (constrained or not) allow us to identify three groups of drawings according to the new metrics. The largest one is the cluster that corresponds to well-performed stars (see Fig. 12 and Table S3), as it gathers the low-density and low-residual drawings. The other two clusters, for any of the metrics, represent the drawings with more errors, although they capture different problems. For all metrics, Cluster 1 indicates a tendency to slightly shortcut the corners, resulting in a smoother path. At the outer corners, the drawing is closer to the inner border, while it approaches the outer border at the inner corners. An increase of density in certain regions, meaning an increase of retracing, highlights that even when the drawing is inside the limits, the participants find specific parts of the figure more cognitively demanding than others. These regions are located close to the corners, where they make a sharp change in the movement. Density manifests the visuomotor planning conflict, meaning that its increase is related to an incorrect movement or hesitation, a common trait found when the task is approached for the first time (Kennedy & Raz, 2005; Lemieux et al., 2014; Miall et al., 2021; Miall & Cole, 2007). When the increase in density is higher, not just localized around the corners, or combined with an increase in the residuals, as represented by Clusters 2 and 3, higher cognitive demands are highlighted, related to a more difficult inhibition of the overlearned responses. This clustering approach is helping us to identify and group distinct drawing patterns, facilitating their interpretation. In our case, the sample corresponds to healthy participants, so no pattern can be associated with any cognitive impairment. However, there are differences in terms of age: Clusters 2 and 3 correspond to older participants. Oscillatory patterns, which would be reflected in an increase of the density, have been previously associated with older age as well (Brosseau et al., 2007), and it has also been seen in patients with Alzheimer’s disease and brain lesions (Rouleau, Décary, et al., 2002a, b; Rouleau, Salmon, et al., 2002a, b). The evolution of such patterns with trials and time can reveal procedural learning difficulties or region-specific deficits.

### Limitations and future perspectives

The proposed method harmonizes data with different setups and shapes. For our analysis, stars coming from online, paper, and app versions were used, which covers a large variety of possibilities. The algorithm is programmed to detect the blue, green, and red colors on the digitized image; it will be extended to correctly identify black and gray and isolate the drawing from the main figure. Although other shapes have been reproduced by our method, only one type of 5-pointed star was used during the tests. Access to other datasets would reinforce its applicability in other shapes.

Another limitation of this study is the sample size and its diversity, which limit the generalization of the results. Increasing the sample size, the number of trials per person, or incorporating a clinical sample, would enhance the conclusions of the research. However, while limited, this sample is useful as an example for the application of the new approach. We chose to use age as a variable of interest, relating to traditional MTT measures, where our results are in line with previous research.

By using the time series approach, we identified different patterns of errors and grouped them into three clusters. We found an age difference between the groups, but the cluster sizes were uneven, so further analysis could not be carried out. Future research may involve larger sample sizes and include older participants to confirm these findings.

Clinical applications of the new method to detect disease-specific patterns and values, as well as tracking maturation, learning, and decline over time, are other possible applications of this method in the future. It will help us identify *where* a certain group of people may struggle more, which corners or turns are more difficult, and therefore understand *why*.

## Conclusion

A new approach to evaluate the Mirror Tracing Task has been proposed in this work using residuals and density. These variables will help data harmonization across different projects, star sizes, or shapes. The summarized version of these variables as a unique value for each star is highly correlated with the number of errors, suggesting that they can replace the current way of analyzing the data. Moreover, the new variables can be studied on an angle- or region-specific basis, providing more insights into the task performance. This latter approach is highly relevant for identifying patterns that can be repeated across participants, groups of people, or even by the same participant across several trials. The analysis as time series-like data opens the possibility for applying their specific statistical approaches to measure similarity between multiple stars. Finally, based on a metric for similarity, a clustering approach or a classification problem is now feasible. Altogether, while time and errors are useful metrics, they summarize the MTT performance in single values, losing its complexity. Our spatially resolved approach *unwraps* the drawing, enabling an analysis focused on the details of each drawing. This approach will help to analyze and harmonize new data from different sources or projects, and it will also enable us to reanalyze old datasets to identify missing features.

## Supplementary Information

Below is the link to the electronic supplementary material.

Supplementary file1 (DOCX 1630 KB)

## Data Availability

The LCBC dataset has restricted access. Requests can be made to the corresponding author, and some of the data can be made available given appropriate ethical and data protection approvals. However, we provide a simulated dataset that replicates many features of the original data to facilitate reproducibility. Additionally, the emulated stars shown at the beginning of the paper are openly available for use. Both the simulated dataset and the emulated drawings are available in https://github.com/PabloFGarrido/ursamirror and have been deposited in the Zenodo repository (Garrido, 2024).

## Footnotes

1 https://projectneuron.illinois.edu/games/mirror-tracing-game-intro.html

2 https://github.com/PabloFGarrido/ursamirror

3 https://github.com/Refridgerator/Wolfram/tree/master

## Code availability

The processing and analysis code can be accessed at: https://github.com/PabloFGarrido/ursamirror and in the Zenodo repository (Garrido, 2024). Most of the code has been collected in a *Python* package named *ursamirror*, which is available in PyPI.

## References

[CR1] Alrubaye, Z., Hudhud Mughrabi, M., Manav, B., & Batmaz, A. U. (2024). Effects of color cues on eye-hand coordination training with a mirror drawing task in virtual environment. *Frontiers in Psychology*. 10.3389/fpsyg.2023.1307590 38288362 10.3389/fpsyg.2023.1307590 PMC10823539

[CR2] Azhari, M. N. S., Rahim, M. N. A. A., Roslan, N. A. S., Mohamed, M. N., Hidayat, Y., & Rahmat, A. (2024). Mirror tracing’s bilateral transfer patterns: Investigating transfer between hemispheres and the function of motor imagery. *Malaysian Journal of Sport Science and Recreation*, *20*(2), Article 2.

[CR3] Backhaus, J., & Junghanns, K. (2006). Daytime naps improve procedural motor memory. *Sleep Medicine,**7*(6), 508–512. 10.1016/j.sleep.2006.04.002 16931152 10.1016/j.sleep.2006.04.002

[CR4] Balslev, D., Christensen, L. O. D., Lee, J.-H., Law, I., Paulson, O. B., & Miall, R. C. (2004). Enhanced accuracy in novel mirror drawing after repetitive transcranial magnetic stimulation-induced proprioceptive deafferentation. *The Journal of Neuroscience,**24*(43), 9698–9702. 10.1523/JNEUROSCI.1738-04.2004 15509758 10.1523/JNEUROSCI.1738-04.2004 PMC6730149

[CR5] Benjamini, Y., & Yekutieli, D. (2001). The control of the false discovery rate in multiple testing under dependency. *The Annals of Statistics,**29*(4), 1165–1188.

[CR6] Bornovalova, M. A., Gratz, K. L., Daughters, S. B., Nick, B., Delany-Brumsey, A., Lynch, T. R., Kosson, D., & Lejuez, C. W. (2008). A multimodal assessment of the relationship between emotion dysregulation and borderline personality disorder among inner-city substance users in residential treatment. *Journal of Psychiatric Research,**42*(9), 717–726. 10.1016/j.jpsychires.2007.07.014 17868698 10.1016/j.jpsychires.2007.07.014

[CR7] Brosseau, J., Potvin, M.-J., & Rouleau, I. (2007). Aging affects motor skill learning when the task requires inhibitory control. *Developmental Neuropsychology,**32*(1), 597–613. 10.1080/87565640701361120 17650996 10.1080/87565640701361120

[CR8] Carlin, J. B., Gurrin, L. C., Sterne, J. A., Morley, R., & Dwyer, T. (2005). Regression models for twin studies: A critical review. *International Journal of Epidemiology,**34*(5), 1089–1099. 10.1093/ije/dyi153 16087687 10.1093/ije/dyi153

[CR9] Corkin, S. (2002). What’s new with the amnesic patient H.M.? *Nature Reviews Neuroscience,**3*(2), 153–160. 10.1038/nrn726 11836523 10.1038/nrn726

[CR10] Dumel, G., Carr, M., Marquis, L.-P., Blanchette-Carrière, C., Paquette, T., & Nielsen, T. (2015). Infrequent dream recall associated with low performance but high overnight improvement on mirror-tracing. *Journal of Sleep Research,**24*(4), 372–382. 10.1111/jsr.12286 25726721 10.1111/jsr.12286

[CR11] Feldman, G., Dunn, E., Stemke, C., Bell, K., & Greeson, J. (2014). Mindfulness and rumination as predictors of persistence with a distress tolerance task. *Personality and Individual Differences,**56*, 154–158. 10.1016/j.paid.2013.08.040 10.1016/j.paid.2013.08.040 PMC3843486 24298196

[CR12] Ferrel-Chapus, C., Hay, L., Olivier, I., Bard, C., & Fleury, M. (2002). Visuomanual coordination in childhood: Adaptation to visual distortion. *Experimental Brain Research,**144*(4), 506–517. 10.1007/s00221-002-1064-2 12037635 10.1007/s00221-002-1064-2

[CR13] Finn, A. S., Kalra, P. B., Goetz, C., Leonard, J. A., Sheridan, M. A., & Gabrieli, J. D. E. (2016). Developmental dissociation between the maturation of procedural memory and declarative memory. *Journal of Experimental Child Psychology,**142*, 212–220. 10.1016/j.jecp.2015.09.027 26560675 10.1016/j.jecp.2015.09.027 PMC4666804

[CR14] Fozard, J. L., Vercruyssen, M., Reynolds, S. L., Hancock, P. A., & Quilter, R. E. (1994). Age differences and changes in reaction time: The Baltimore Longitudinal Study of Aging. *Journal of Gerontology,**49*(4), P179–P189. 10.1093/geronj/49.4.P179 8014399 10.1093/geronj/49.4.p179

[CR15] Gabrieli, J. D. E., Corkin, S., Mickel, S. F., & Growdon, J. H. (1993). Intact acquisition and long-term retention of mirror-tracing skill in Alzheimer’s disease and in global amnesia. *Behavioral Neuroscience,**107*(6), 899–910. 10.1037/0735-7044.107.6.899 8136066 10.1037//0735-7044.107.6.899

[CR16] Garrido, P. F. (2024). *UrsaMirror* (Version v0.2.3) [Computer software]. Zenodo. 10.5281/zenodo.13987806

[CR17] Gielis, J. (2003). A generic geometric transformation that unifies a wide range of natural and abstract shapes. *American Journal of Botany,**90*(3), 333–338. 10.3732/ajb.90.3.333 21659124 10.3732/ajb.90.3.333

[CR18] Gullaud-Toussaint, L., & Vinter, A. (2003). The effect of discordant sensory information in graphic production: Two distinct subject groups. *Psychological Research,**67*(4), 291–302. 10.1007/s00426-002-0129-y 14634816 10.1007/s00426-002-0129-y

[CR19] Harris, C. R., Millman, K. J., van der Walt, S. J., Gommers, R., Virtanen, P., Cournapeau, D., Wieser, E., Taylor, J., Berg, S., Smith, N. J., Kern, R., Picus, M., Hoyer, S., van Kerkwijk, M. H., Brett, M., Haldane, A., del Río, J. F., Wiebe, M., Peterson, P., & Oliphant, T. E. (2020). Array programming with NumPy. *Nature,**585*(7825), 357–362. 10.1038/s41586-020-2649-2 32939066 10.1038/s41586-020-2649-2 PMC7759461

[CR20] Harrison, N. A., Doeller, C. F., Voon, V., Burgess, N., & Critchley, H. D. (2014). Peripheral inflammation acutely impairs human spatial memory via actions on medial temporal lobe glucose metabolism. *Biological Psychiatry,**76*(7), 585–593. 10.1016/j.biopsych.2014.01.005 24534013 10.1016/j.biopsych.2014.01.005 PMC4166523

[CR21] Hunter, J. D. (2007). Matplotlib: A 2D graphics environment. *Computing in Science & Engineering,**9*(3), 90–95. 10.1109/MCSE.2007.55

[CR22] Julius, M. S., & Adi-Japha, E. (2016). A developmental perspective in learning the Mirror-Drawing Task. *Frontiers in Human Neuroscience*, *10*. https://www.frontiersin.org/articles/10.3389/fnhum.2016.00083 10.3389/fnhum.2016.00083 PMC4773595 26973498

[CR23] Kennedy, K. M., & Raz, N. (2005). Age, sex and regional brain volumes predict perceptual-motor skill acquisition. *Cortex,**41*(4), 560–569. 10.1016/S0010-9452(08)70196-5 16042032 10.1016/s0010-9452(08)70196-5

[CR24] Lemieux, L. G., Simoneau, M., Tessier, J.-F., Billot, M., Blouin, J., & Teasdale, N. (2014). Balance control interferes with the tracing performance of a pattern with mirror-reversed vision in older persons. *AGE,**36*(2), 823–837. 10.1007/s11357-013-9601-4 24258770 10.1007/s11357-013-9601-4 PMC4039253

[CR25] Mantua, J., Baran, B., & Spencer, R. M. C. (2016). Sleep benefits consolidation of visuo-motor adaptation learning in older adults. *Experimental Brain Research,**234*(2), 587–595. 10.1007/s00221-015-4490-7 26563162 10.1007/s00221-015-4490-7 PMC6398605

[CR26] McKinney, W. (2010). *Data Structures for Statistical Computing in Python*. 56–61. 10.25080/Majora-92bf1922-00a

[CR27] Miall, R. C., Afanasyeva, D., Cole, J. D., & Mason, P. (2021). The role of somatosensation in automatic visuo-motor control: A comparison of congenital and acquired sensory loss. *Experimental Brain Research,**239*(7), 2043–2061. 10.1007/s00221-021-06110-y 33909112 10.1007/s00221-021-06110-y PMC8282580

[CR28] Miall, R. C., & Cole, J. (2007). Evidence for stronger visuo-motor than visuo-proprioceptive conflict during mirror drawing performed by a deafferented subject and control subjects. *Experimental Brain Research,**176*(3), 432–439. 10.1007/s00221-006-0626-0 16874511 10.1007/s00221-006-0626-0

[CR29] Milner, B., Squire, L. R., & Kandel, E. R. (1998). Cognitive neuroscience and the study of memory. *Neuron,**20*(3), 445–468. 10.1016/S0896-6273(00)80987-3 9539121 10.1016/s0896-6273(00)80987-3

[CR30] Nissen, C., Kloepfer, C., Nofzinger, E. A., Feige, B., Voderholzer, U., & Riemann, D. (2006). Impaired sleep-related memory consolidation in primary insomnia—a pilot study. *Sleep,**29*(8), 1068–1073. 10.1093/sleep/29.8.1068 16944676 10.1093/sleep/29.8.1068

[CR31] Petitjean, F., Ketterlin, A., & Gançarski, P. (2011). A global averaging method for dynamic time warping, with applications to clustering. *Pattern Recognition,**44*(3), 678–693. 10.1016/j.patcog.2010.09.013

[CR32] Project NEURON. (n.d.). *Mirror Tracing Game*. Retrieved September 5, 2024, from https://neuron.illinois.edu/games/mirror-tracing-game-intro.html

[CR33] Rasch, B., Pommer, J., Diekelmann, S., & Born, J. (2009). Pharmacological REM sleep suppression paradoxically improves rather than impairs skill memory. *Nature Neuroscience,**12*(4), 396–397. 10.1038/nn.2206 18836440 10.1038/nn.2206

[CR34] Rawn, K. P., & Keller, P. S. (2023). Child emotion lability is associated with within-task changes of autonomic activity during a mirror-tracing task. *Psychophysiology,**60*(10), Article e14354. 10.1111/psyp.14354 37246804 10.1111/psyp.14354

[CR35] Renna, M. E., Chin, S., Seeley, S. H., Fresco, D. M., Heimberg, R. G., & Mennin, D. S. (2018). The use of the mirror tracing persistence task as a measure of distress tolerance in generalized anxiety disorder. *Journal of Rational-Emotive & Cognitive-Behavior Therapy,**36*(1), 80–88. 10.1007/s10942-017-0274-2

[CR36] Rodrigue, K. M., Kennedy, K. M., & Raz, N. (2005). Aging and longitudinal change in perceptual-motor skill acquisition in healthy adults. *The Journals Of Gerontology Series B: Psychological Sciences And Social Sciences,**60*(4), P174–P181. 10.1093/geronb/60.4.P174 15980284 10.1093/geronb/60.4.p174

[CR37] Romanowska, S., & Best, M. W. (2023). Examining the role of failure and success experiences on task persistence and neurocognition in schizophrenia-spectrum disorders. *Journal of Clinical and Experimental Neuropsychology,**45*(3), 255–269. 10.1080/13803395.2023.2227406 37357679 10.1080/13803395.2023.2227406

[CR38] Rouleau, I., Décary, A., Chicoine, A.-J., & Montplaisir, J. (2002). Procedural skill learning in obstructive sleep apnea syndrome. *Sleep,**25*(4), 398–408. 10.1093/sleep/25.4.398 12071541

[CR39] Rouleau, I., Salmon, D. P., & Vrbancic, M. (2002). Learning, retention and generalization of a mirror tracing skill in Alzheimer’s disease. *Journal of Clinical and Experimental Neuropsychology,**24*(2), 239–250. 10.1076/jcen.24.2.239.997 11992206 10.1076/jcen.24.2.239.997

[CR40] Sakoe, H., & Chiba, S. (1978). Dynamic programming algorithm optimization for spoken word recognition. *IEEE Transactions on Acoustics, Speech, and Signal Processing,**26*(1), 43–49. 10.1109/TASSP.1978.1163055

[CR41] Salowitz, N. M. G., Eccarius, P., Karst, J., Carson, A., Schohl, K., Stevens, S., Van Hecke, A. V., & Scheidt, R. A. (2013). Brief report: Visuo-spatial guidance of movement during gesture imitation and mirror drawing in children with autism spectrum disorders. *Journal of Autism and Developmental Disorders,**43*(4), 985–995. 10.1007/s10803-012-1631-8 22898762 10.1007/s10803-012-1631-8

[CR42] Schloss, H. M., & Haaga, D. A. F. (2011). Interrelating behavioral measures of distress tolerance with self-reported experiential avoidance. *Journal of Rational-Emotive & Cognitive-Behavior Therapy,**29*(1), 53–63. 10.1007/s10942-011-0127-3 21448252 10.1007/s10942-011-0127-3 PMC3064486

[CR43] Schultz, D., & Jain, B. (2018). Nonsmooth analysis and subgradient methods for averaging in dynamic time warping spaces. *Pattern Recognition,**74*, 340–358. 10.1016/j.patcog.2017.08.012

[CR44] Scoville, W. B., & Milner, B. (1957). Loss of recent memory after bilateral hippocampal lesions. *Journal of Neurology, Neurosurgery & Psychiatry,**20*(1), 11–21. 10.1136/jnnp.20.1.11 13406589 10.1136/jnnp.20.1.11 PMC497229

[CR45] Seabold, S., & Perktold, J. (2010). statsmodels: Econometric and statistical modeling with Python. *Proceedings of the 9th Python in Science Conference*. 10.25080/Majora-92bf1922-011

[CR46] Sokol, E. (n.d.). *Wolfram/Кaк нapиcoвaть звeздy (и нe тoлькo) в пoляpныx кoopдинaтax.nb at master · Refridgerator/Wolfram*. GitHub. Retrieved October 25, 2024, from https://github.com/Refridgerator/Wolfram/tree/master

[CR47] Taghian, N. R., Parsons, E. M., Fitzgerald, H. E., Zvolensky, M. J., Gorlin, E. I., Doan, S., & Otto, M. W. (2025). Stressful life events and depression in adolescents from low-income neighborhoods: An investigation of the role of working memory capacity and distress intolerance. *Cognitive Therapy And Research,**49*(1), 169–176. 10.1007/s10608-024-10510-z

[CR48] Tavenard, R., Faouzi, J., Vandewiele, G., Divo, F., Androz, G., Holtz, C., Payne, M., Yurchak, R., Rußwurm, M., Kolar, K., & Woods, E. (2020). Tslearn, a machine learning toolkit for time series data. *Journal of Machine Learning Research,**21*(118), 1–6.34305477

[CR49] The pandas development team. (2024). *pandas-dev/pandas: Pandas* (Version v2.2.1) [Computer software]. Zenodo. 10.5281/zenodo.10697587

[CR50] Veilleux, J. C., Pollert, G. A., Zielinski, M. J., Shaver, J. A., & Hill, M. A. (2019). Behavioral assessment of the negative emotion aspect of distress tolerance: Tolerance to emotional images. *Assessment,**26*(3), 386–403. 10.1177/1073191116689819 28135808 10.1177/1073191116689819

[CR51] Vicari, S., Finzi, A., Menghini, D., Marotta, L., Baldi, S., & Petrosini, L. (2005). Do children with developmental dyslexia have an implicit learning deficit? *Journal of Neurology, Neurosurgery & Psychiatry,**76*(10), 1392–1397. 10.1136/jnnp.2004.061093 16170083 10.1136/jnnp.2004.061093 PMC1739378

[CR52] Virtanen, P., Gommers, R., Oliphant, T. E., Haberland, M., Reddy, T., Cournapeau, D., Burovski, E., Peterson, P., Weckesser, W., Bright, J., van der Walt, S. J., Brett, M., Wilson, J., Millman, K. J., Mayorov, N., Nelson, A. R. J., Jones, E., Kern, R., Larson, E., & van Mulbregt, P. (2020). SciPy 1.0: Fundamental algorithms for scientific computing in Python. *Nature Methods,**17*(3), 261–272. 10.1038/s41592-019-0686-2 32015543 10.1038/s41592-019-0686-2 PMC7056644

[CR53] Waldrop, D., Kumar, M., Kumar, A. M., Wilkie, F. L., & Eisdorfer, C. (2001). Mirror star-tracing performance in HIV-1 infection: An assessment of visuoconstructive skills? *Stress and Health,**17*(5), 287–290. 10.1002/smi.910

[CR54] Walt, Svander, Schönberger, J. L., Nunez-Iglesias, J., Boulogne, F., Warner, J. D., Yager, N., Gouillart, E., & Yu, T. (2014). Scikit-image: Image processing in Python. *PeerJ,**2*, Article e453. 10.7717/peerj.453 25024921 10.7717/peerj.453 PMC4081273

[CR55] Waskom, M. L. (2021). Seaborn: Statistical data visualization. *Journal of Open Source Software,**6*(60), Article 3021. 10.21105/joss.03021

[CR56] Zhang, Z., Tavenard, R., Bailly, A., Tang, X., Tang, P., & Corpetti, T. (2017). Dynamic time warping under limited warping path length. *Information Sciences,**393*, 91–107. 10.1016/j.ins.2017.02.018

