# Supplementary material for: Unwrapping the mirror tracing task
Source: Behav Res Methods. 2026 Mar 5;58(3):80. doi: 10.3758/s13428-025-02845-6 (PMC12963139; doi:10.3758/s13428-025-02845-6)
Supplement: Supplementary file 1 — Supplementary file1 (DOCX 1630 KB) [file 13428_2025_2845_MOESM1_ESM.docx]

**Supplementary Information for:**

**Unwrapping the Mirror Tracing Task**

Pablo F. Garrido^1,2^, Anne Cecilie Sjøli Bråthen^1^, Emilie Sogn Falch^1^, Jonas Kransberg^1^, Anders M. Fjell^1,3^, Øystein Sørensen^1^ and Kristine B. Walhovd^1,3^

^1^Center for Lifespan Changes in Brain and Cognition, Department of Psychology, University of Oslo, Oslo, Norway.

^2^Department of Physics, University of Oslo, Oslo, Norway.

^3^Computational Radiology and Artificial Intelligence, Department of Radiology and Nuclear Medicine, Oslo University Hospital, Oslo, Norway.

Figure S1

*Examples of figures with different number of peaks using the proposed equation*


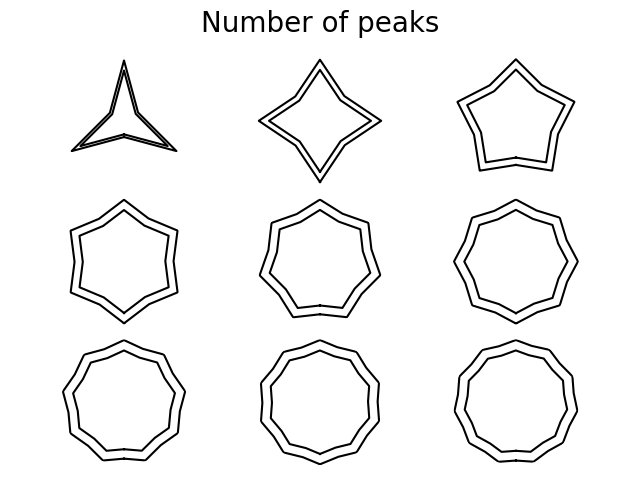


*Note.* Variations of the MTT star that can be analyzed by our method, obtained through Equation 1. The size, side deepness, width, and peak smoothness are kept constant, while the number of peaks varies from 3 to 11.

Figure S2

*Examples of figures with different side deepness using the proposed equation*


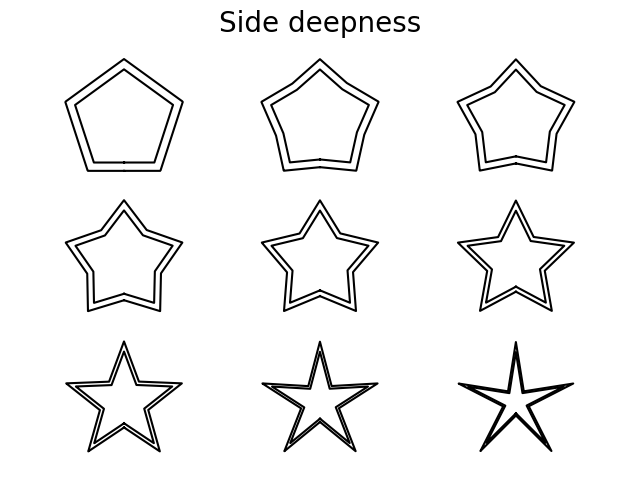


*Note.* Variations of the MTT star that can be analyzed by our method, obtained through Equation 1. The size, number of peaks, width, and peak smoothness are kept constant, while side deepness (parameter *m*) varies from 1 to 3.5.

Figure S3

*Examples of figures with different border width using the proposed equation*


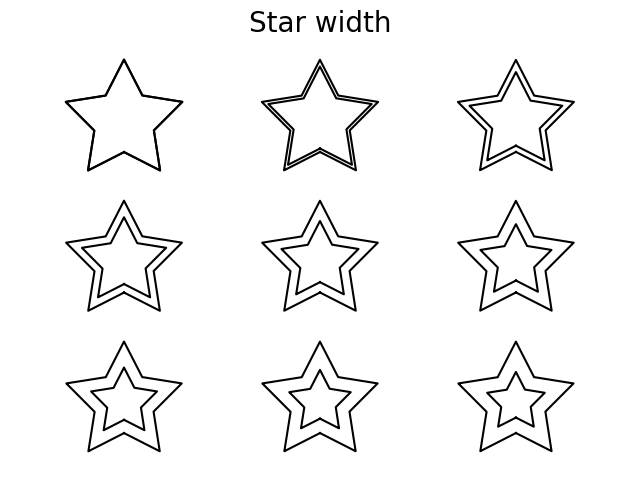


*Note.* Variations of the MTT star that can be analyzed by our method, obtained through Equation 1. The size, number of peaks, side deepness, and peak smoothness are kept constant, while star width, measured as the relative difference between the outer and inner radius varied from 0 to 1 (same size to double size).

Figure S4

*Examples of figures with different peak smoothness using the proposed equation*


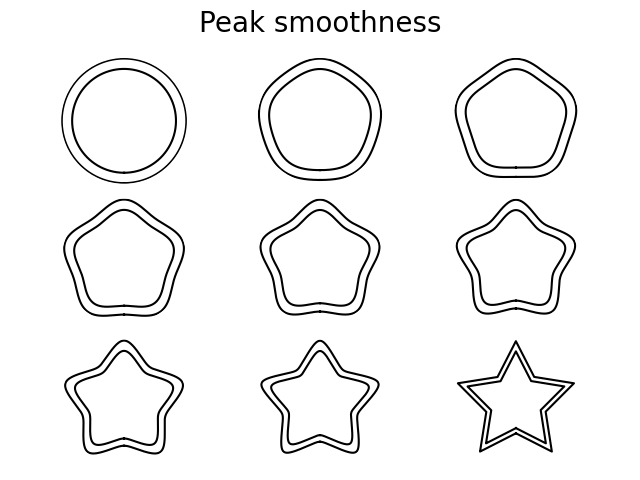


*Note.* Variations of the MTT star that can be analyzed by our method, obtained through Equation 1. The size, number of peaks, side deepness, and width are kept constant, while peak smoothness, parameter *k*, varies from 0 to 1 (from completely round to sharp).

Figure S5

*Density and residuals distributions for the 4 emulated stars*


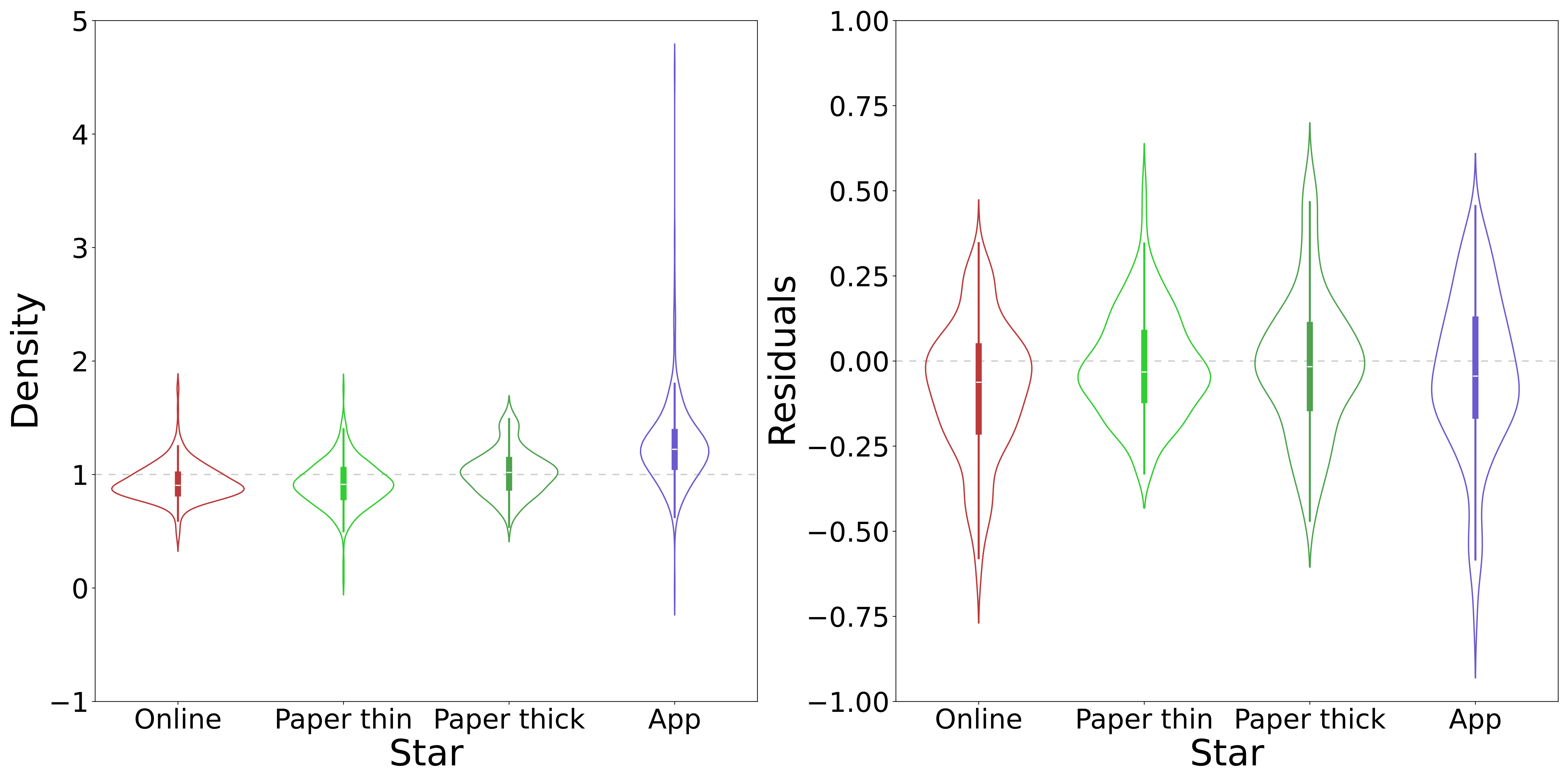


*Note.* Violin plots representing the distribution of each variable and containing a boxplot inside for complementary information. The median is shown with a white mark inside the boxplot. Left: Density values distribution, determined for every 1º, for the Online, Paper thin, Paper thick, and App drawing examples. The dashed line represents the Density=1 value, corresponding to the ideal star drawing. A mean value lower than 1 is usually related to a drawing closer to the inner border than in the middle of the lines. Right: Residuals values distribution, determined for every 1º, for the same examples. The dashed line represents the Residuals=0 value, corresponding to the ideal star drawing.

Figure S6

*Mean density and residuals for the 4 emulated stars*


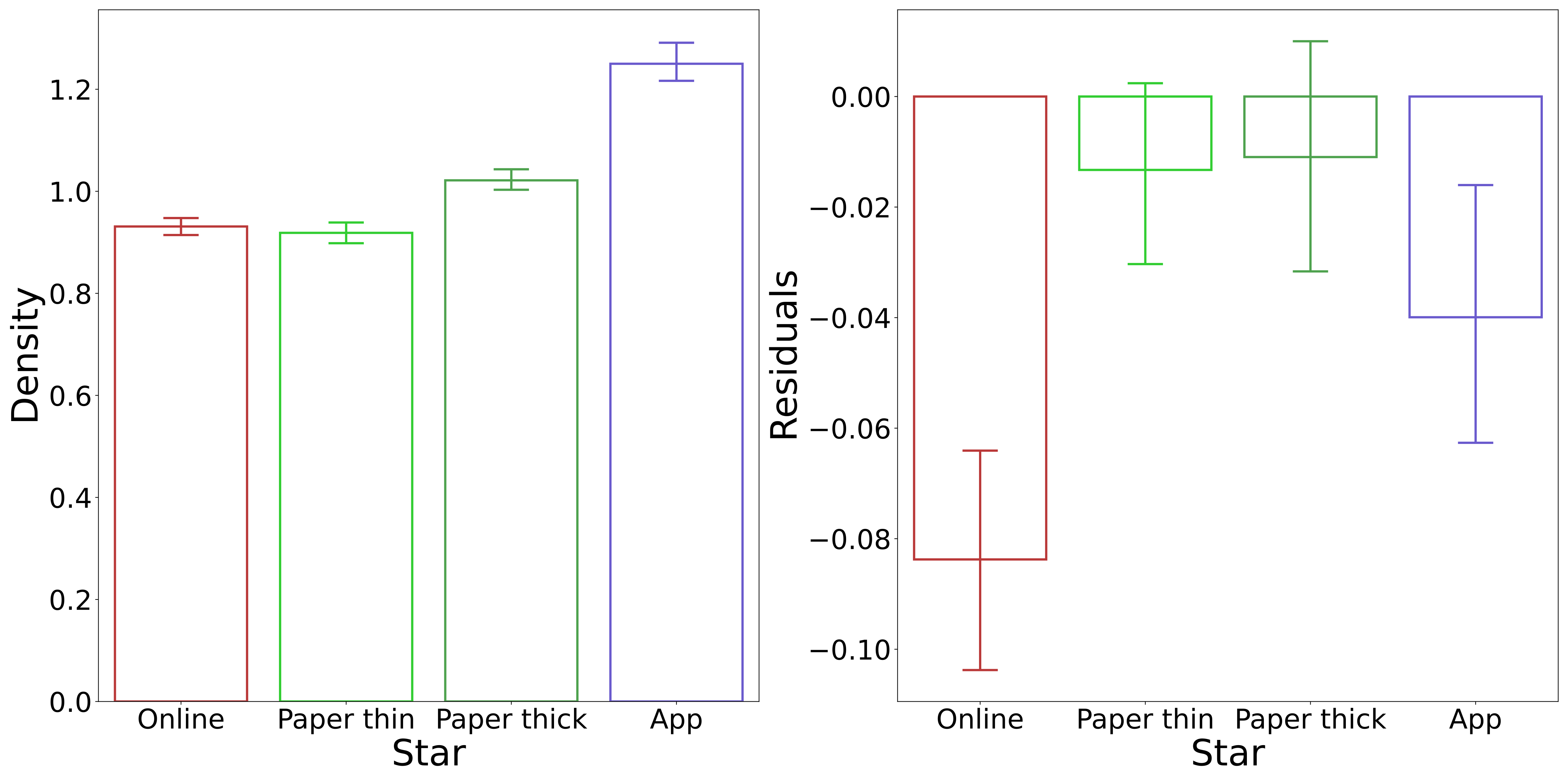


*Note.* Barplots representing the mean value of each magnitude for the Online, Paper thin, Paper thick, and App drawing examples. The error bars correspond to 1.96 times the standard error of the mean around its value. Left: Density mean values. Paper thick values are slightly higher than the Paper thin due to the change in the marker, even though the harmonization tries to reduce these differences. The App mean value is higher due to the error in the left side of the drawing. Right: Residuals mean values. Both Paper drawings’ means are quite similar, although the Online and App have more negative values.

Table S1

*Summarized parameters for the 4 emulated stars*

|  | $\bar{\rho}$ | $\sigma\left( \bar{\rho} \right)$ | $IQR\left( \rho\right)$ | $\bar{\Delta r^{'}}$ | $\sigma\left( \bar{\Delta r^{'}} \right)$ | $IQR\left( \Delta r^{'} \right)$ |
| --- | --- | --- | --- | --- | --- | --- |
| Online | 0.9310 | 0.0085 | 0.1666 | -0.084 | 0.010 | 0.251 |
| Paper thin | 0.919 | 0.010 | 0.241 | -0.0133 | 0.0082 | 0.1978 |
| Paper thick | 1.021 | 0.010 | 0.245 | -0.011 | 0.011 | 0.245 |
| App | 1.250 | 0.019 | 0.308 | -0.040 | 0.012 | 0.283 |

*Note.* Mean, standard error of the mean, and interquartile range (IQR) of the Density ($\rho$) and Residuals ($\Delta r^{'}$) for the Online, Paper thin, Paper thick and App drawing examples.

Figure S7

*Density and residuals distributions for the well-performed and anomalous stars*


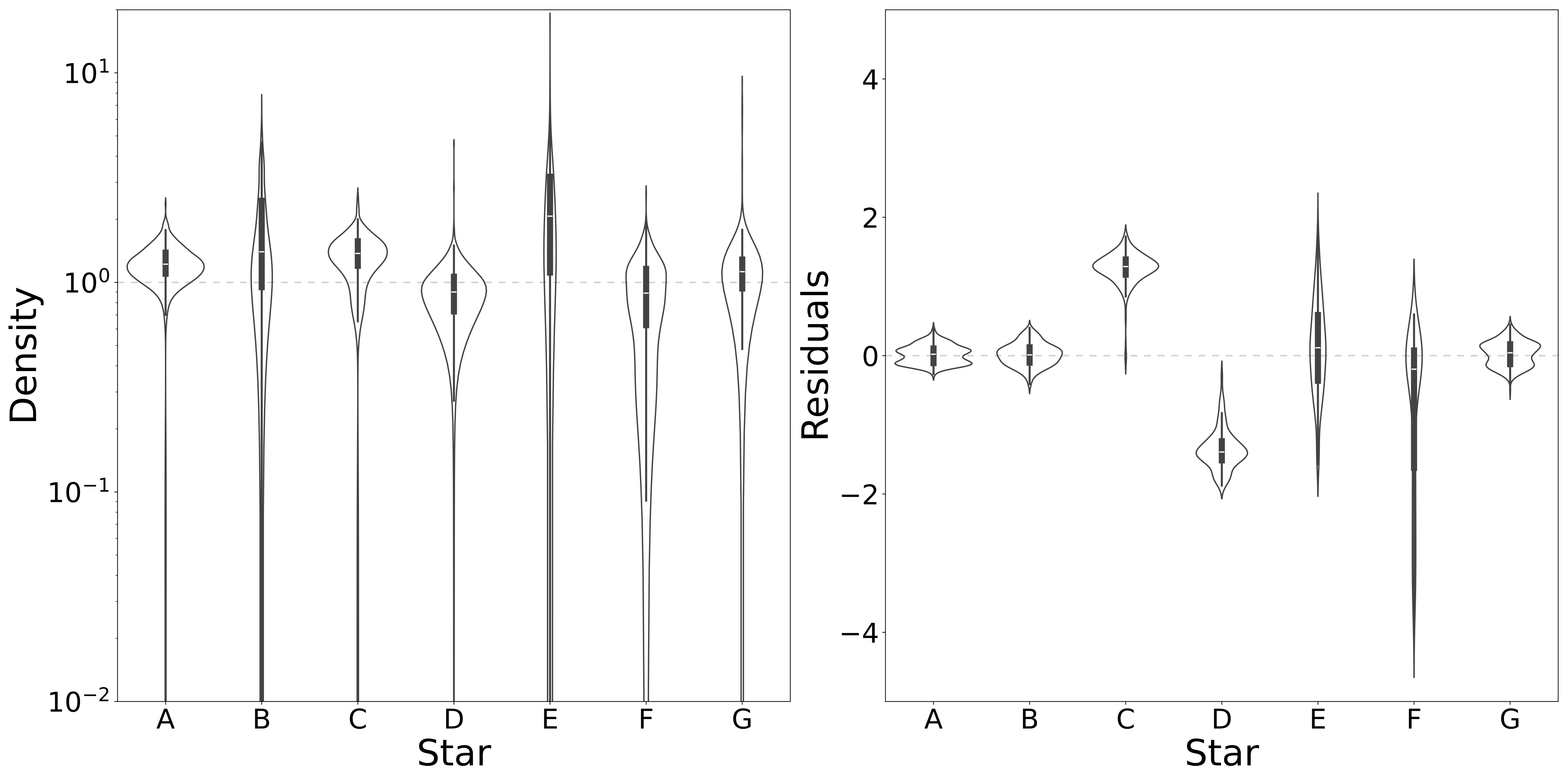


*Note.* Violin plots representing the distribution of each variable and containing a boxplot inside for complementary information. The median is shown with a white mark inside the boxplot. Left: Density values distribution, on a logarithmic scale, determined for every 1º, for the well-performed and anomalous stars examples. The dashed line represents the Density=1 (10^0^) value, corresponding to the ideal star drawing. Stars B and E correspond to oscillatory patterns, which increases the range of the Density values’ distribution. Star F has larger tail towards low values, as it reflects the drawing with shortcuts. Right: Residuals values distribution, determined for every 1º, for the same examples. The dashed line represents the Residuals=0 value, corresponding to the ideal star drawing. Stars C and D correspond to the drawings completely out of the figure, being highly positive (outside of the drawing to the exterior) or negative (outside, towards the center). The oscillatory pattern with high amplitude (star E) is reflected in a wider Residuals distribution. Star F has a large tail on the negative values due to the shortcut.

Figure S8

*Mean density and residuals for the well-performed and anomalous stars*


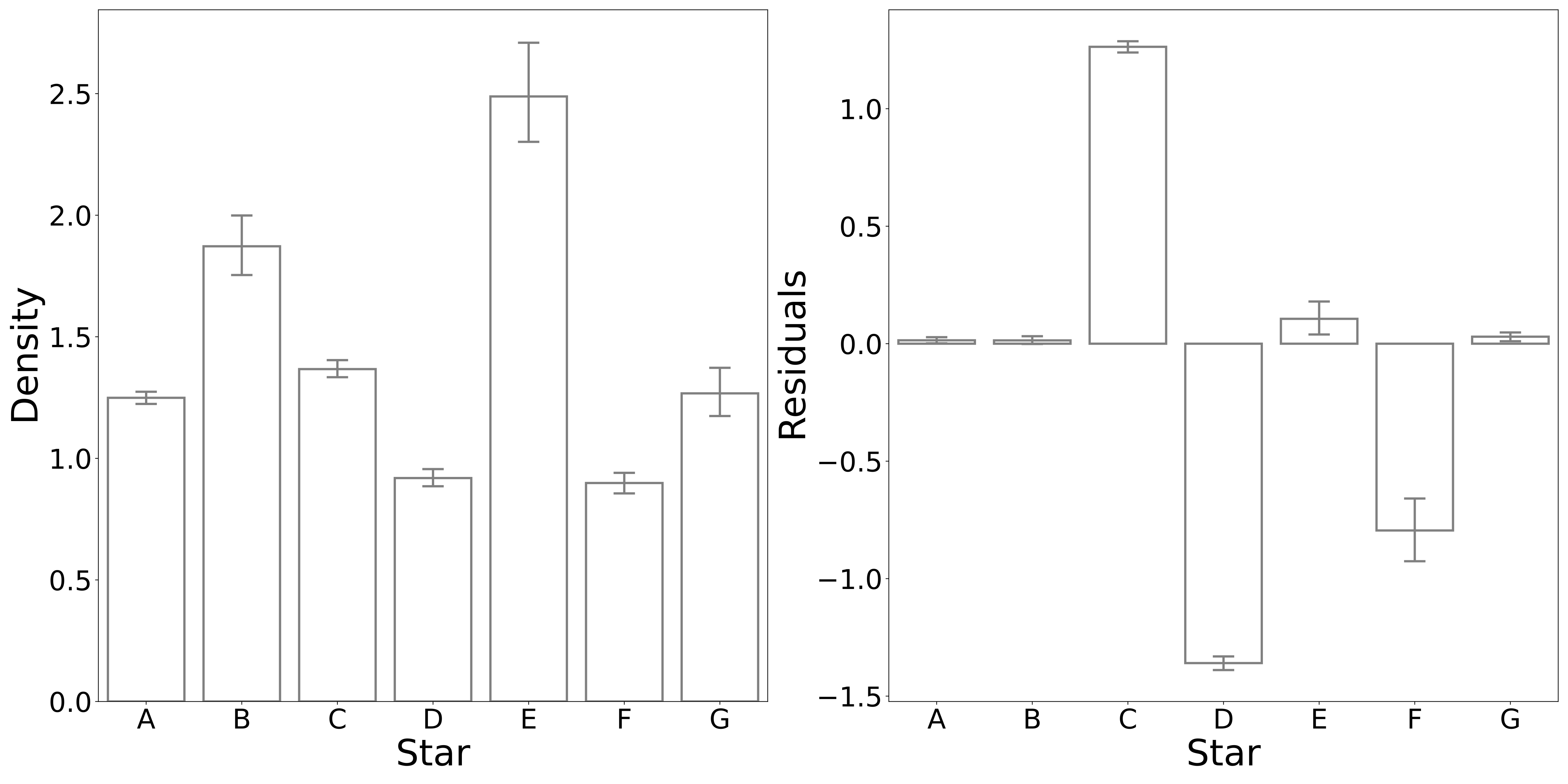


*Note.* Barplots representing the mean value of each magnitude for the well-performed and anomalous stars examples. The error bars correspond to 1.96 times the standard error of the mean around its value. Left: Density mean values. Using the well-performed star (A) as a reference, the oscillatory drawings (B and C) have larger density values; star D (completely outside, towards the center) and F (the drawing with shortcuts) have lower values; star C (completely outside, towards the exterior side) has slightly larger density as more traces or pixels are needed to trace the whole drawing; and star G, which contains a located error, has similar mean value as star A, but larger standard error. Right: Residuals mean values. Stars C and D, corresponding to those drawings completely out of the figure, have the larger residuals, followed by star F (the drawing with shortcuts). The oscillatory drawings have different mean values and standard errors, with star E values larger than those from B, due to the amplitude of its oscillations.

Table S2

*Summarized parameters for the well-performed and anomalous stars*

|  | $\bar{\rho}$ | $\sigma\left( \bar{\rho} \right)$ | $IQR\left( \rho\right)$ | $\bar{\Delta r^{'}}$ | $\sigma\left( \bar{\Delta r^{'}} \right)$ | $IQR\left( \Delta r^{'} \right)$ |
| --- | --- | --- | --- | --- | --- | --- |
| Star A | 1.249 | 0.013 | 0.287 | 0.0144 | 0.0070 | 0.2123 |
| Star B | 1.872 | 0.064 | 1.504 | 0.0141 | 0.0086 | 0.2220 |
| Star C | 1.367 | 0.018 | 0.371 | 1.264 | 0.012 | 0.220 |
| Star D | 0.919 | 0.018 | 0.337 | -1.359 | 0.015 | 0.277 |
| Star E | 2.49 | 0.10 | 2.08 | 0.106 | 0.037 | 0.950 |
| Star F | 0.899 | 0.021 | 0.535 | -0.795 | 0.064 | 1.697 |
| Star G | 1.267 | 0.052 | 0.350 | 0.0298 | 0.0093 | 0.2890 |

*Note.* Mean, standard error of the mean, and interquartile range (IQR) of the Density ($\rho$) and Residuals ($\Delta r^{'}$) for the well-performed (A) and the anomalous drawing examples.

Figure S9

*Regression coefficients distribution for Time, Residuals, Density and Errors*


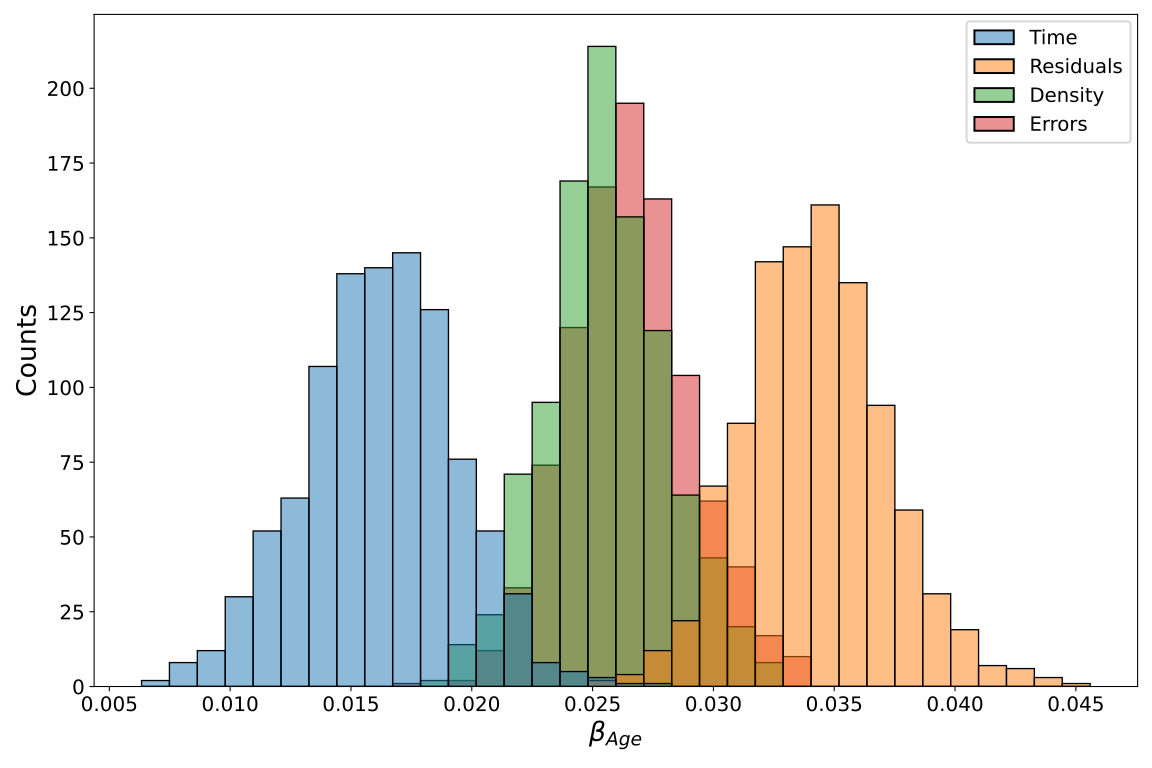


*Note.* Distribution of the *β* coefficients for Z-scored Time (blue), Residuals (orange), Density (green), and Errors (red), determined on each iteration of the bootstrap method.

Table S3

*Number of participants included in each cluster for every metric used in the unsupervised clustering.*

|  | Cluster 1 | Cluster 2 | Cluster 3 |
| --- | --- | --- | --- |
| ED | 171 | 25 | 14 |
| c-DTW | 173 | 26 | 11 |
| DTW | 183 | 5 | 22 |

Figure S10

*Group age and sex comparison for each cluster and every used metric*


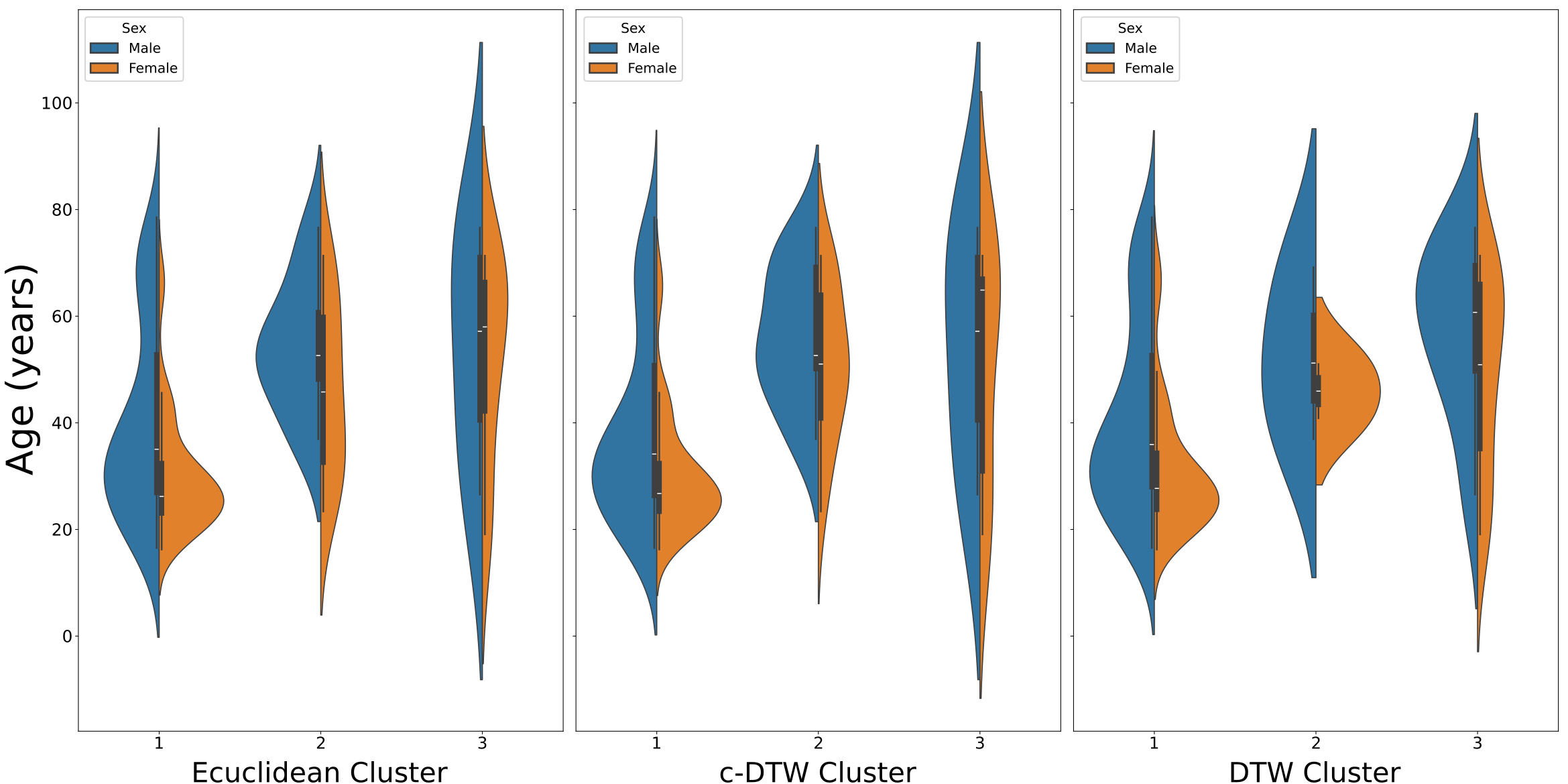
 *Note.* Violin plots representing the distribution of age for each drawing cluster and containing a boxplot inside for complementary information. The median is shown with a white mark inside the boxplot. The distributions are divided by sex, with blue representing Male and orange representing Female. From left to right: Euclidean, c-DTW (constrained Dynamic Time Warping), and DTW (Dynamic Time Warping) are the metrics used for the unsupervised clustering of the drawings. The best drawings (fewer errors) are always clustered in the first group, corresponding to a younger sample.
